# The role of CA1 CB1 receptors on lithium-induced spatial memory impairment in rats

**DOI:** 10.17179/excli2018-1511

**Published:** 2018-09-20

**Authors:** Salar Vaseghi, Vahab Babapour, Mohammad Nasehi, Mohammad-Reza Zarrindast

**Affiliations:** 1 Department of Physiology, Faculty of Veterinary Science, Science and Research Branch, Islamic Azad University, Tehran, Iran; 2 Department of Basic Sciences, Faculty of Veterinary Medicine, University of Tehran, Tehran, Iran; 3 Cognitive and Neuroscience Research Center (CNRC), Amir-Almomenin Hospital, Tehran Medical Sciences Branch, Islamic Azad University, Tehran, Iran; 4 Department of Pharmacology School of Medicine, Tehran University of Medical Sciences, Tehran, Iran; 5 Institute for Cognitive Science Studies (ICSS), Tehran, Iran; 6 Department of Neuroendocrinology, Endocrinology and Metabolism Research Institute, Tehran University of Medical Sciences, Tehran, Iran

**Keywords:** ACPA, AM251, lithium, spatial memory, CA1, rats

## Abstract

Lithium, a glycogen synthase kinase-3β (GSK-3β) inhibitor, prevents cannabinoid withdrawal syndrome, but there is limited data exploring the interaction between lithium and cannabinoid system on memory processes. The present study aimed to test the interaction between dorsal hippocampal (CA1 region) cannabinoid system and lithium on spatial memory in rats. Spatial memory was assessed in Morris Water Maze (MWM) apparatus by a single training session of eight trials. The results showed that pre-training intra-CA1 microinjection of ACPA, the cannabinoid type 1 receptor (CB1r) agonist, at doses of 0.001, 0.01 or 1 µg/rat, or AM251, the cannabinoid type 1 receptor (CB1r) antagonist, at doses of 1, 10 or 100 ng/rat, increased escape latency and traveled distance to the platform, suggesting a spatial learning impairment, whereas intraperitoneal administration of lithium (0.5, 1 or 5 mg/kg) had no effect on spatial learning. Also, rats that received lithium plus a lower dose of ACPA (0.001 µg/rat) or AM251 (1 ng/rat) had successful performance in the MWM. In the probe test, the results showed that pre-training administration of lithium (5 mg/kg) and ACPA (0.01 or 1 µg/rat) but not AM251 (at all doses used) impaired spatial memory retrieval. Also, lower dose of ACPA (0.001 µg/rat) or AM251 (1 ng/rat) potentiated the effect of ineffective doses of lithium (0.5 and 1 mg/kg) on spatial memory retrieval, while restored the effect of effective dose of lithium (5 mg/kg). In conclusion, cannabinoids may have a dual effect on lithium-induced spatial memory impairment in rats.

## Introduction

Lithium is a primary drug for management and treatment of bipolar disorder (Malhi et al., 2012[48]). It regulates signal transduction pathways in different regions of the brain, and changes the function of several neurotransmitter systems involved in memory processing (Parsaei et al., 2016[60]). Glycogen Synthase Kinase-3β (GSK-3β) inhibited rapidly by lithium through different pathways (Beurel and Jope, 2006[5]; Jope, 2003[30]). GSK-3β has a critical role in the CNS and is expressed in neurons during their different processes of remodeling and regulating (Dill et al., 2008[14]; Manji et al., 1999[49]). There are several side effects of lithium that impact different body tissues (Malhi et al., 2009[47]). Lithium impairs the ability of learning and memory, and decreases the speed of information processing (Albert et al., 2014[1]). Previous studies showed that post-training or pre-test administration of lithium in the dorsal hippocampal (CA1) region impairs memory of inhibitory avoidance task (Ghorbanalizadeh-Khalifeh-Mahaleh et al., 2008[23]), and impairs spatial memory in the Morris Water Maze (MWM) apparatus (Parsaei et al., 2016[60]). 

The hippocampus has a critical role in spatial learning and memory (Erickson and Barnes, 2003[20]). Studies with functional Magnetic Resonance Imaging (fMRI) and Positron Emission Tomography (PET) have revealed that the level of activation in the hippocampus is greater in tasks which require spatial memory (Kumaran and Maguire, 2005[38]; Maguire et al., 1997[46]). The hippocampus of rats and other animals represents their environments, locations and their contents (Burgess et al., 2002[6]). Neurological damage in the hippocampus has been associated with impairment in the spatial performance and the ability to navigate (Teng and Squire, 1999[78]). The dorsal hippocampal (CA1) region has a main role in spatial memory because lesions in this region lead to severe impairments in the formation of spatial memory, whereas lesions in the ventral hippocampus have not been shown to produce similar impairment (Pothuizen et al., 2004[64]). 

The CA1 region has numerous interneurons expressing cannabinoid type 1 receptor (CB1r) (Jappy et al., 2016[28]). CB1r is widely expressed in the different CNS regions including the hippocampus, amygdala, cerebellum and the cortex (Pertwee and Ross, 2002[63]). CB1r activation by Tetrahydrocannabinol (THC) and synthetic cannabinoids impairs learning and memory function (Takahashi et al., 2005[76]). In the MWM apparatus, THC impairs spatial learning and memory by activating CB1r, because pre-treatment with SR141716A (CB1r antagonist) prevents spatial memory deficit (Da and Takahashi, 2002[11]). Also, it has been revealed that cannabinoids, as is the case with lithium, may inhibit GSK3-β (Ozaita et al., 2007[57]).

As we mentioned above, lithium and cannabinoids impair various aspects of cognitive performance, including learning and memory. Also, we know that these drugs modulate some neurotransmitter systems specific to memory processing, and further that, they can inactivate GSK-3β. Thus, for these similar effects, lithium and cannabinoids may have interactions in some different experiments. Previous studies have shown that cannabinoids, such as THC and Cannabidiol (CBD) suppress lithium-induced vomiting, while their effect is linear and biphasic, respectively (Parker et al., 2004[59]). In other research, AM251 potentiated lithium-induced conditioned gaping in rats (Limebeer et al., 2010[43]). Also, it has been revealed that lithium attenuates cannabinoid-induced dependence in rats, while this effect probably is related to the ERK1/2 & GSK-3β pathways (Rahimi et al., 2014[66]). 

One of the side effects of lithium and cannabinoids is impairment across different types of memory. The memory impairment effects of cannabinoids are mediated by CB1r. Given the role of CB1r in the CA1 region, and the fact that this region has an important role in spatial memory, it is necessary to explicate the exact impact of CA1 CB1 receptors on spatial memory. A better understanding of this relationship will lead to a better understanding of how lithium and cannabinoids affect similar neurotransmitters and GSK-3β. Taken together, the primary goal of the current study is to establish the impact of the interaction between lithium and cannabinoids on spatial memory.

## Material and Methods

### Animals 

Male Wistar rats weighing 220-240 g were obtained from Institute for Cognitive Sciences studies (ICSS), Tehran, Iran. All animals were maintained five per Plexiglas cage in a room. The room was kept at a stable temperature (22 ± 2 °C), humidity (40-60 %) and a 12/12 light-dark cycle, lights on at 7:00 a.m. Rats were allowed to get acclimatized to the lab environment for 7 days before starting experiments. Also they had *ad libitum* access to food and water except during the experiments. All experimental schemes were carried out during the light phase between 8:00 and 17:00. We tried to reduce the number of animals used and their suffering. The experimental protocol in this study was approved by the Research and Ethics Committee of the School of Advanced Technologies in Medicine, Tehran University of Medical Sciences and were done in accordance with the National Institutes of Health Guide for the Care and Use of Laboratory Animals (NIH publications No. 80-23).

### Stereotaxic surgery

All animals were anesthetized by intraperitoneal injection of a ketamine and xylazine (100 and 10 mg/kg, respectively) mixture. After administration, rats were placed in a stereotaxic apparatus (Stoelting Co., Wood Dale, IL, USA). Two steel guide cannulae (22-guage) were bilaterally implanted into the CA1 region of the dorsal hippocampus (AP: -3.3 mm from bregma; ML: ± 2 mm from midline; DV: 2.8 mm from the skull surface) according to Paxinos and Watson's atlas (Paxinos and Watson, 2007[61]). The guide cannulae were secured by jeweler's screws, and the incision was closed with rapidly polymerizing dental acrylic cement. After surgery, all animals were allowed to recover for 7 days. This period was necessary for rats to getting clear from the anesthetic effects. 

### Drugs preparation and administration procedures

Lithium chloride (Daroopakhsh, Tehran, Iran) was dissolved in normal saline and administrated intraperitoneally (i.p.). The selective CB1r antagonist, AM251, and the selective CB1r agonist, ACPA, were stored at -14 °C. Doses of 1, 10 and 100 ng of AM251 and doses of 0.001, 0.01 and 1 µg of ACPA were dissolved in anhydrous ethanol and were diluted by normal saline to achieve their required doses (Khodayar et al., 2016[33]; Najar et al., 2015[54]). The bilateral microinjections of AM251 and ACPA into the CA1 region (intra-CA1) were done by using a 2 µl Hamilton syringe. This syringe was connected to an injection needle (27 guage) by a polyethylene tube. The injection needle was inserted 1 mm beyond the tip of the guide cannula. Infusions were delivered in volume of 0.5 µl during 60 s into each side. 

### Apparatus and behavioral procedure

#### Apparatus

All rats were trained in the Morris Water Maze, a valid apparatus for evaluation spatial memory in rodents (Vorhees and Williams, 2006[79]). This apparatus is a circular black tank, 150 cm in diameter and 60 cm deep, filled with water to a depth of 30 cm. The water temperature was maintained at 20 ± 2 ºC. Different visual cues were placed on the walls of the water maze room and their position was stable during the experiments. The maze was divided into four equal quadrants. Each quadrant had starting location called north (N), south (S), west (W) and east (E) at equal distances on the rim. A hidden platform (10 cm in diameter) was submerged 1 cm beneath the surface of the water in the center of target quadrant (the north-west quadrant). During the experiments, the animal motion was recorded by a camera located above the maze which was connected to a computer. A video tracking system (Etho-Vision XTv 8.5; Noldus Information Technology, Wageningen, the Netherlands) was used to measure the parameters. In the trial session, parameters such as escape latency (time to find the hidden platform), traveled distance (path length to find the hidden platform) and swimming speed (swimming velocity of rat in each training session) were measured. In the probe session, the time spent and the distance traveled in each quadrant were measured.

#### Behavioral procedure

The single training session included eight trials with four different starting positions. Each of the starting positions were equally distributed around the perimeter of the maze. Each trial was started by placing an animal in one of the quadrants. The swimming time was 60 s and each rat was allowed to find the hidden platform during this time. Distal spatial cues guided rats to find the hidden platform. After finding the hidden platform, rats were allowed to remain on the platform for 20 s to memorized spatial cues and situation. Then they were placed in a cage for 20 s until the start of the next trial. If a rat did not find the platform during 60 s, it was manually guided to the platform by the researcher and allowed to rest for 20 s. In each trial, three parameters were evaluated; escape latency (s), traveled distance (cm) and swimming speed (cm/s). For measuring development of spatial memory, we used the escape latency and the distance to platform results, in such a way that the longer the distance traveled and the time spent to find a platform, means the greater memory impairment. Swimming speed was used to evaluate motor functions. Probe trial (retrieval test session) was carried out 24 h after the training day. Before starting the probe trial, we removed the hidden platform from the tank. The probe trial consisted of a 60 s free swimming period, with spent time and the traveled distance in the target quadrant recorded for measuring spatial memory retrieval, considered to indicate greater memory impairment. For non-spatial visible test evaluation, the platform was elevated 2 cm above the water and covered with a piece of aluminum foil in the center of the north-east quadrant. This test was performed after the probe trial. This procedure is believed to provide information on the possible non-specific effects involving motor, visual, or motivational abilities unrelated to learning and memory. 

### Experimental design

#### Effects of lithium on spatial memory

This experiment was carried out to evaluate the effects of pre-training administration of lithium on spatial memory acquisition/retrieval. Four groups of animals received saline (1 ml/kg) or lithium (0.5, 1 and 5 mg/kg, i.p.) 5 min before training (pre-training administration). After administration, animals were placed in the Morris Water Maze for training. 

#### Effects of intra-CA1 microinjection of ACPA or AM251 on spatial memory

This experiment was carried out to evaluate the effects of pre-training administration of ACPA or AM251 on spatial memory acquisition/retrieval. Four groups of animals received vehicle (1 µl/rat; two groups), ACPA (0.001, 0.01 and 1 µg/rat, intra-CA1) or AM251 (1, 10 and 100 ng/rat, intra-CA1) 5 min before training (pre-training administration). After administration, animals were placed in the Morris Water Maze for training. 

#### Effects of intra-CA1 microinjection of ACPA or AM251 on lithium-induced spatial memory impairment

This experiment was carried out to evaluate the effects of intra-CA1 microinjection of ACPA or AM251 during lithium-induced spatial memory impairment. Eight groups of animals received subthreshold dose of ACPA (0.001 µg/rat, intra-CA1; four groups) or AM251 (1 ng/rat, intra-CA1; four groups) 5 min after administration of saline (1 µl/rat) or three different doses of lithium (0.5, 1 and 5 mg/kg, i.p.). 5 min after the second injection animals were placed in the Morris Water Maze for training. 

### Statistical analysis

#### The trial session

Obtained data were analyzed using a two-way analysis of variance (ANOVA) considering the trials as the repeated measures factor and treatments as between-subjects factor. In combination studies, the results were analyzed using a three-way ANOVA with AM251 and lithium between-subject factors and the trials as the within-subject factor, and also, with ACPA and lithium between-subject factors and the trials as the within-subject factor.

#### The probe session

Obtained data were analyzed with two-way ANOVA with treatment as between-subject factor and quadrant as within-subject factor. Post-hoc Tukey test was used for within-subject comparisons. In all comparisons, the P-values 0.05 and less were considered as statistically significant.

## Results

### Effects of intra-CA1 microinjection of ACPA on spatial learning and swimming speed

Repeated measure analysis on spatial learning showed that for both escape latency and traveled distance, the main effect of dose [(F_3,28_ = 32.83, P < 0.001) and (F_3,28_ = 39.37, P < 0.001), respectively], the effect of trial [(F_3.87,108.44_ = 409.32, P < 0.001) and (F_4.51,126.31_ = 426.98, P < 0.001), respectively] and interaction effect [(F_11.62,108.44_ = 3.55, P < 0.001) and (F_13.53,126.31_ = 5.22, P < 0.001), respectively] were significant. Post hoc comparisons demonstrated that ACPA at all administered doses increased escape latency and traveled distance (trials 3 to 8). While, ACPA increased both only at doses of 0.01 and 1 µg/ rat (trial 2). 

**Figure 1:**
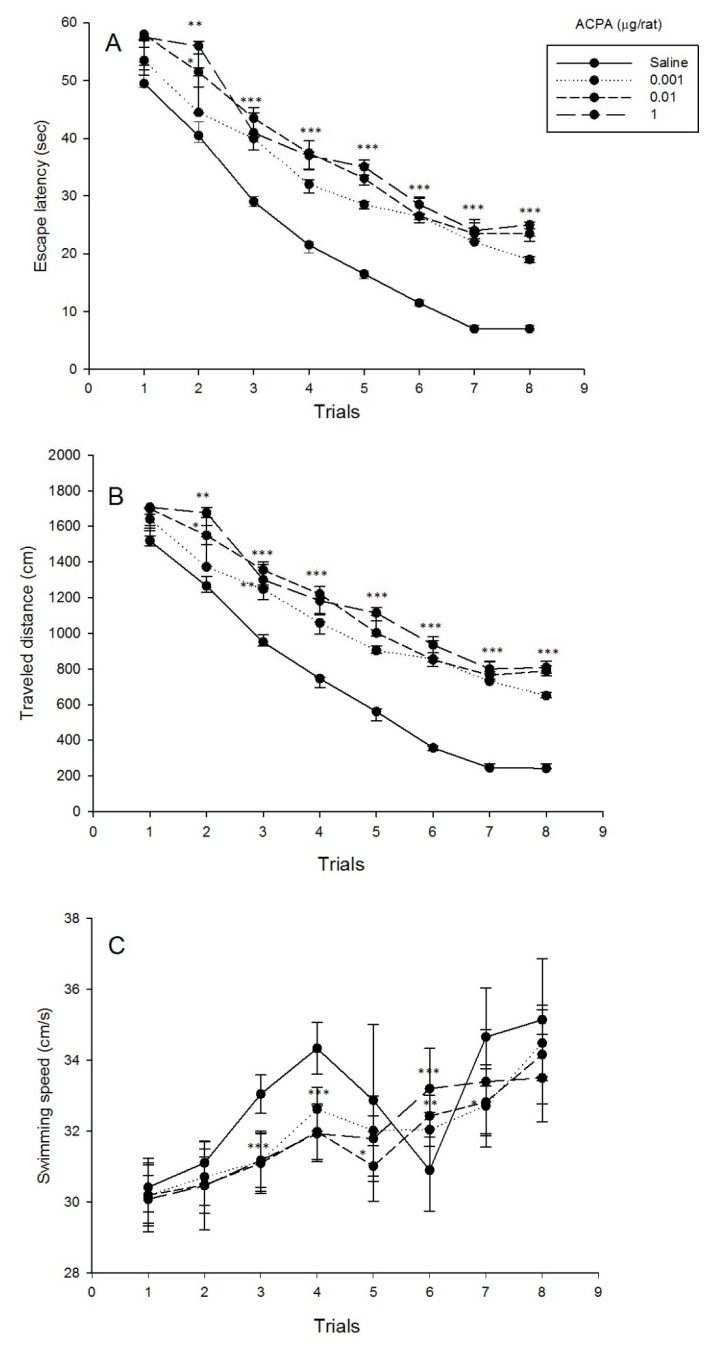
Effects of ACPA on spatial learning and swimming speed. Four groups of eight animals received pre-training intra-CA1 administration of saline (1 µl/rat) or different doses of ACPA (0.001, 0.01 and 1 µg/rat). 5 min after the injection animals were trained in MWM apparatus. Escape latency, traveled distance and swimming speed across eight trials were shown. Values are presented as mean ± S.E.M for each experimental group. *P<0.5, **P < 0.01 and ***P < 0.001 different from the saline control group. (For all raw data of this Figure see Supplementary Table 1).

Additionally, repeated measure analysis on swimming speed showed that the main effect of dose (F_3,28_ = 5.74, P < 0.01), the effect of trial (F_4.90,137.28_ = 65.52, P < 0.001) and interaction effect (F_14.71,137.28_ = 4.25, P < 0.001) were significant. Post hoc comparisons demonstrated that ACPA at all administered doses decreased swimming speed (trials 3 and 4). Additional analyses indicated that ACPA decreased swimming speed only at dose of 0.01 µg/rat (trial 5), ACPA decreased swimming speed only at doses of 0.001 and 0.01 µg/rat (trial 7), and finally that ACPA increased swimming speed only at doses of 0.01 and 1 µg/rat (trial 6) (Figure 1(Fig. 1), see also Supplementary data).

### Effects of intra-CA1 microinjection of AM251 on spatial learning and swimming speed

Repeated measure analysis on spatial learning showed that for both escape latency and traveled distance, the main effect of dose [(F_3,28_ = 75.27, P < 0.001) and (F_3,28_ = 75.13, P < 0.001), respectively], the effect of trial [(F_4.12,115.45_ = 370.69, P < 0.001) and (F_4.34,121.56_ = 328.35, P < 0.001), respectively] and interaction effect [(F_12.37,115.45_ = 1.92, P < 0.05) and (F_13.02,121.56_ = 2.38, P < 0.01), respectively] were significant. Post hoc comparisons demonstrated that AM251 at all administered doses increased escape latency and traveled distance (trials 3 to 8), and further that AM251 increased escape latency only at doses of 10 and 100 ng/rat (trial 2). 

**Figure 2:**
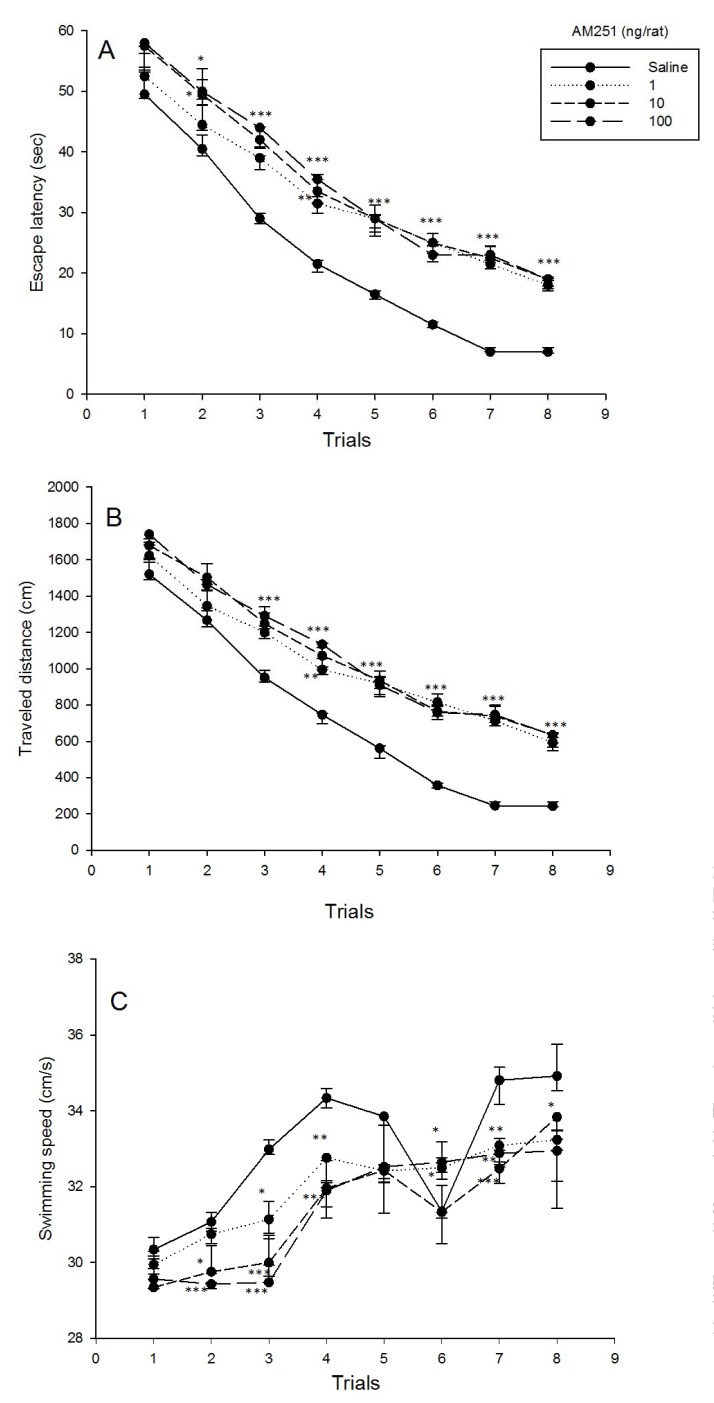
Effects of AM251 on spatial learning and swimming speed. Four groups of eight animals received pre-training intra-CA1 administration of saline (1 µl/rat) or different doses of AM251 (1, 10 and 100 ng/rat). 5 min after the injection animals were trained in MWM apparatus. Escape latency, traveled distance and swimming speed across eight trials were shown. Values are presented as mean ± S.E.M for each experimental group. *P < 0.5, **P < 0.01 and ***P < 0.001 different from the saline control group. (For all raw data of this Figure see Supplementary Table 2).

Also repeated measure analysis on swimming speed showed that the main effect of dose (F_3,28_ = 19.14, P < 0.001), the effect of trial (F_4.73,132.44_ = 38.51, P < 0.001) and interaction effect (F_14.19,132.44_ = 3.20, P < 0.001) were significant. Post hoc comparisons demonstrated that AM251 at all administered doses decreased swimming speed (trials 3, 4 and 7). While, AM251 decreased swimming speed only at doses of 10 and 100 ng/rat (trial 2). Also AM251 decreased swimming speed only at dose of 10 ng/rat (trial 8). But AM251 increased swimming speed at doses of 1 and 100 ng/rat (trial 6) (Figure 2(Fig. 2), see also Supplementary data).

### Effects of interactions between lithium and intra-CA1 microinjection of ACPA or AM251 on spatial learning and swimming speed

#### Effect of lithium, by itself

Repeated measure analysis on spatial learning showed that for both escape latency and traveled distance, the effect of trial [(F_3.26,91.21_ = 333.24, P < 0.001) and (F_3.84,107.64_ = 350.45, P < 0.001), respectively] was significant. While the other effects such as the main effect of dose and interaction effect did not alter. Also repeated measure analysis on swimming speed showed that the main effect of dose (F_3,28_ = 12.81, P < 0.001), the effect of trial (F_4.07,114.10_ = 16.82, P < 0.001) and interaction effect (F_12.22,114.10_ = 4.22, P < 0.001) were significant. Post hoc comparisons demonstrated that lithium at all administered doses decreased swimming speed (trial 8), whereas lithium decreased swimming speed only at dose of 5 mg/kg (trials 1, 3 and 7).

#### Effect of interaction between ACPA and lithium

Three way repeated measure ANOVA analysis on spatial learning showed that for both escape latency and traveled distance, the main effect of ACPA dose [(F_1,56_ = 42.34, P < 0.001) and (F_1,56_ = 56.53, P < 0.001), respectively], the main effect of lithium dose [(F_3,56_ = 7.61, P < 0.001) and (F_3,56_ = 9.89, P < 0.001), respectively], the main effect of interaction between lithium and ACPA doses [(F_3,56_ = 14.93, P < 0.001) and (F_3,56_ = 15.94, P < 0.001), respectively], the effect of trial [(F_3.32,185.74_ = 727.26, P < 0.001) and (F_3.85,215.83_ = 773.40, P < 0.001), respectively] and interaction between trial, lithium and ACPA [(F_9.95,185.74_ = 1.91, P < 0.05) and (F_11.56,215.83_ = 2.46, P < 0.01), respectively] were significant. Also, the effect of lithium doses (F_11.56,215.83_ = 2.25, P < 0.05) was significant only for traveled distance. Post hoc comparisons demonstrated that ACPA increased both escape latency and traveled distance only at dose of 0.5 mg/kg of lithium (trial 3). Also, three way repeated measure ANOVA analysis on swimming speed showed that the main effect of lithium dose (F_3,56_ = 7.16, P < 0.001), interaction between the main effect of lithium and ACPA dose (F_3,56_ = 8.23, P < 0.001), the effect of trial (F_4.91,275.00_ = 41.40, P < 0.001), the effect of lithium (F_14.73,275.00_ = 5.52, P < 0.001) and interaction effect (F_14.73,275.00_ = 1.77, P < 0.05) were significant. Post hoc comparisons demonstrated that ACPA increased swimming speed at dose of 5 mg/kg of lithium (trial 3).

#### Effect of interaction between AM251 and lithium

**Figure 3:**
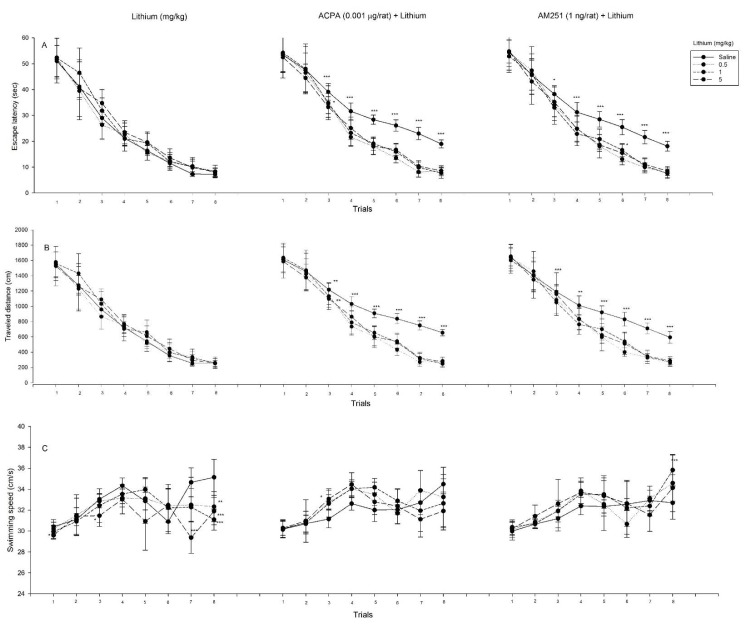
Effects of lithium, and interaction between cannabinoids and lithium on spatial learning and swimming speed. The animals received pre-training intraperitoneal administration of saline (1 ml/kg) or different doses of lithium (0.5, 1 and 5 mg/kg). 5 min after pervious injection the animals received lower dose of ACPA (0.001 µg/rat; four groups, eight rats in each group) or AM251 (1 ng/rat; four groups, eight rats in each group). Escape latency, traveled distance and swimming speed across eight trials were shown. Values are presented as mean ± S.E.M for each experimental group. *P < 0.5, **P < 0.01 and ***P < 0.001 different from the respective control group. (For all raw data of this Figure see Supplementary Table 3).

Three way repeated measure ANOVA analysis on spatial learning showed that for both escape latency and traveled distance, the main effect of AM251 dose [(F_1,56_ = 35.85, P < 0.001) and (F_1,56_ = 43, P < 0.001), respectively], the main effect of lithium dose [(F_3,56_ = 4.74, P < 0.01) and (F_3,56_ = 5.79, P < 0.01), respectively], the main effect of interaction between lithium and AM251 doses [(F_3,56_ = 10.85, P < 0.001) and (F_3,56_ = 10.43, P < 0.001), respectively] and the effect of trial [(F_3.66,204.99_ = 749.99, P < 0.001) and (F_4.32,241.79_ = 753.27, P < 0.001), respectively] were significant. Also the effect of lithium doses (F_12.95,241.79_ = 2.19, P < 0.05) and interaction between trial, lithium and AM251 (F_12.95,241.79_ = 2.08, P < 0.05) were significant only for traveled distance. Post hoc comparisons demonstrated that AM251 increased only escape latency at dose of 1 mg/kg of lithium (trial 6). Also three way repeated measure ANOVA analysis on swimming speed showed that the main effect of lithium dose (F_3,56_ = 2.94, P < 0.05), interaction between the main effect of lithium and AM251 doses (F_3,56_ = 12.95, P < 0.001), the effect of trial (F_5.10,285.59_ = 41.84, P < 0.001), AM251 (F_5.10,285.59_ = 3.61, P < 0.01), lithium (F_15.30,285.59_ = 2.83, P < 0.001) and interaction effect (F_15.30,285.59_ = 3.56, P < 0.001) were significant. Post hoc comparisons demonstrated that AM251 increased swimming speed at dose of 1 mg/kg of lithium (trial 8) (Figure 3(Fig. 3), see also Supplementary data).

### Effects of lithium, ACPA and AM251 on spatial memory retrieval

#### Effect of lithium

For the probe test, one-way ANOVA analysis for escape latency and traveled distance evaluation showed that for both time and distance, the effect of lithium [(F_3,28_ = 13.84, P < 0.001) and (F_3,28_= 34.62, P < 0.001), respectively] was significant. Post hoc comparisons demonstrated that lithium at dose of 5 mg/kg decreased both escape latency and traveled distance in the target quadrant. These results indicating that lithium impaired spatial memory retrieval. For the visible test, one-way ANOVA analysis for escape latency and traveled distance evaluation showed that for both time and distance, the effect of lithium was not significant.

#### Effect of ACPA

For the probe test, one-way ANOVA analysis for escape latency and traveled distance evaluation showed that for both time and distance, the effect of ACPA [(F_3,28_ = 17.44, P < 0.001) and (F_3,28_ = 29.60, P < 0.001), respectively] was significant. Post hoc comparisons demonstrated that ACPA at doses of 0.01 and 1 µg/rat decreased both escape latency and traveled distance in the target quadrant. These results indicate that ACPA impaired spatial memory retrieval. For the visible test, one-way ANOVA analysis for escape latency and traveled distance evaluation showed that for both time and distance, the effect of ACPA was not significant.

#### Effect of AM251

**Figure 4:**
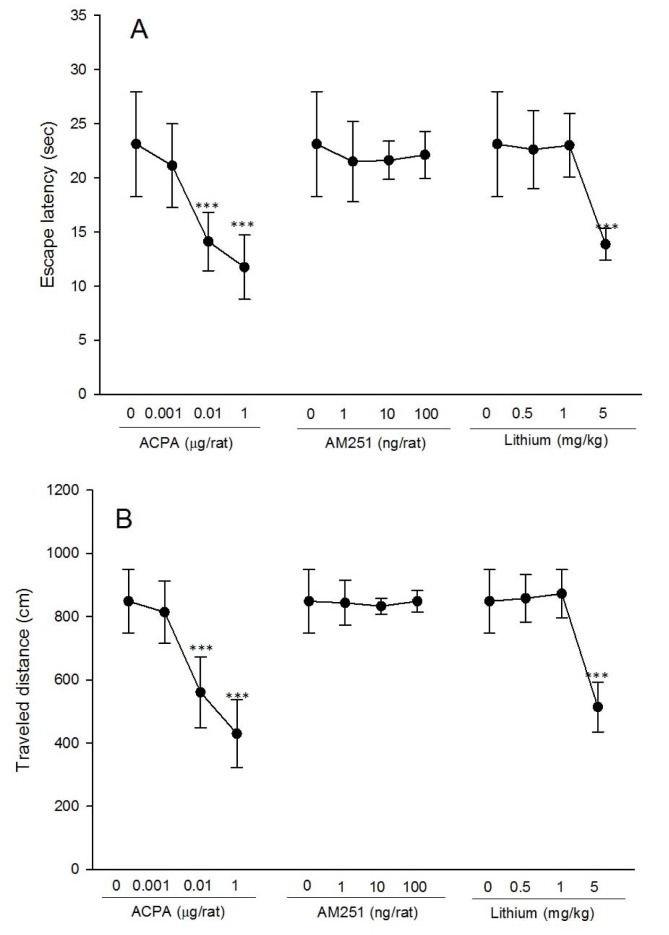
Effects of different doses of all drugs (ACPA, AM251 and lithium) on spatial memory retrieval were shown. Twenty-four hours after training, all animals (eight rats in each group), were trained for the probe test in MWM apparatus and the time spent and the traveled distance in the target were measured. Values are presented as mean ± S.E.M per group. *P < 0.5, **P < 0.01 and ***P < 0.001 different from the saline control group. (For all raw data of this Figure see Supplementary Table 4).

For the probe test, one-way ANOVA analysis for escape latency and traveled distance evaluation showed that for both time and distance, the effect of AM251 was not significant. In other words, AM251 did not alter spatial memory retrieval. For the visible test, one-way ANOVA analysis for escape latency and traveled distance evaluation showed that for both time and distance, the effect of AM251 was not significant (Figure 4(Fig. 4), see also Supplementary data).

### Effects of intra-CA1 microinjection of ACPA or AM251 on lithium-induced spatial memory impairment

#### Effect of interaction between ACPA and lithium on spatial memory retrieval

For the probe test, two-way ANOVA analysis for escape latency and traveled distance evaluation showed that for both time and distance, the effect of ACPA [(F_1,56_ = 26.42, P < 0.001) and (F_1,56_ = 30.16, P < 0.001), respectively], lithium [(F_3,56_ = 10.24, P < 0.001) and (F_3,56_ = 14.06, P < 0.001), respectively] and interaction effect [(F_3,56_ = 20.42, P < 0.001) and (F_3,56_ = 39.68, P < 0.001), respectively] were significant. Post hoc comparisons demonstrated that lower dose of ACPA (0.001 µg/rat) potentiated the spatial memory impairment effect of lithium (0.5 and 1 mg/kg). While, restored the spatial memory impairment effect of lithium (5 mg/ kg). In general, ACPA induced a dual effect in response to ineffective and effective doses of lithium on spatial memory retrieval. For the visible test, two-way ANOVA analysis for escape latency and traveled distance evaluation showed that for both time and distance, the interaction effect of ACPA and lithium was not significant.

#### Effect of interaction between AM251 and lithium on spatial memory retrieval

**Figure 5:**
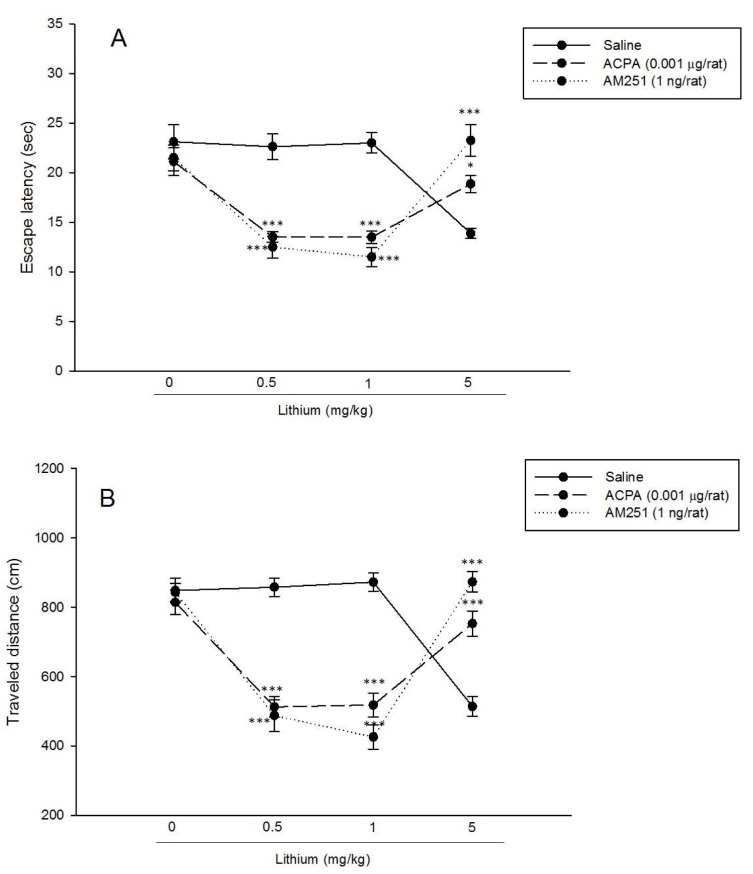
Effects of interaction between cannabinoid drugs and lithium on spatial memory retrieval were shown. Twenty-four hours after training, all animals (eight rats in each group), were trained for the probe test in MWM apparatus and the time spent and the traveled distance in the target were measured. Values are presented as mean ± S.E.M per group. *P < 0.5, **P < 0.01 and ***P < 0.001 different from the respective control group. (For all raw data of this Figure see Supplementary Table 5).

For the probe test, two-way ANOVA analysis for escape latency and traveled distance evaluation showed that for both time and distance, the effect of AM251 [(F_1,56_ = 15.51, P < 0.001) and (F_1,56_ = 25.84, P < 0.001), respectively], lithium [(F_3,56_ = 6.99, P < 0.001) and (F_3,56_ = 15.27, P < 0.001), respectively] and interaction effect [(F_3,56_ = 29.77, P < 0.001) and (F_3,56_ = 66.30, P < 0.001), respectively] were significant. Post hoc comparisons demonstrated that ineffective dose of AM251 (1 ng/rat) potentiated the spatial memory impairment effect of lithium (0.5 and 1 mg/kg), while, restored the spatial memory impairment effect of lithium (5 mg/kg). In general, AM251 induced a dual effect in response to ineffective and effective doses of lithium on spatial memory retrieval. For the visible test, two-way ANOVA analysis for escape latency and traveled distance evaluation showed that for both time and distance, the interaction effect of AM251 and lithium was not significant (Figure 5(Fig. 5), see also Supplementary data).

## Discussion

### Spatial memory impairment effect of lithium

In all experimental groups, lithium was not associated with impairment on spatial learning and acquisition (Figure 3A, B(Fig. 3)); however, lithium was associated with impaired spatial memory retrieval (Figure 4(Fig. 4)), and it decreased swimming speed in some trials (Figure 3C(Fig. 3)). Lithium is used for long-term treatment of bipolar disorder, and has a powerful effect for the prevention of manic/depressive recurrences (Geddes et al., 2010[22]). The clear mechanism of lithium remains unknown (Misztal et al., 2017[52]), but lithium can inhibit GSK3-β by increasing phosphorylation on the key serine residue (Ser9) (Jope, 2003[30]; Klein and Melton, 1996[35]). GSK3-β inhibition induced by lithium is an important contribution to its mood stabilizing therapeutic effects (Jope, 2011[29]). Glycogen Synthase Kinase (GSK), is a kinase that phosphorylates and inactivates glycogen synthase (Embi et al., 1980[19]), and it has important roles in several cell functions (Jope and Johnson, 2004[31]). The activity of GSK3 is regulated by phosphorylation on a regulatory serine (serine-21 in GSK3-α and serine-9 in GSK3-β) and is inhibited by phosphorylation of these regulatory serines. Many of the GSK3-β targets are involved in pathways which are related to cell growth, apoptosis, learning and memory (Kaidanovich-Beilin and Woodgett, 2011[32]). Although lithium has many therapeutic effects on learning, memory and neurological disorders such as Down syndrome, Fragile X syndrome and Alzheimer's disease (AD) (Contestabile et al., 2013[10]; Liu and Smith, 2014[44]; Ly et al., 2013[45]; Nunes et al., 2007[56]), there are important side effects that must be considered including the inducement of dementia and other cognitive impairments (Dunn et al., 2005[17]; Weingartner et al., 1985[80]; Wingo et al., 2009[81]). Administration of lithium and other GSK3-β inhibitors may have impairment effect on memory and other cognitive performances (Stip et al., 2000[73]; Wingo et al., 2009[81]). However, the effect of GSK3-β inhibition on learning and memory is still inconsistent (Chew et al., 2015[8]). A previous study showed that GSK3-β was inhibited during Long-Term Potentiation (LTP), but was activated during Long-Term Depression (LTD) (Peineau et al., 2007[62]). Also, GSK3-β may be involved in memory reconsolidation in the hippocampus, because the genetic reduction and pharmacological inhibition of GSK3-β impaired reconsolidation of hippocampus-dependent place memory. However, the mechanism of memory formation and consolidation in the hippocampus may involve consonant activities in the other brain regions (Kimura et al., 2008[34]). On the other hand, glutamate, an abundant excitatory neurotransmitter in the brain, activates various receptors involved in synaptic plasticity, learning and memory (Collingridge and Bliss, 1995[9]). The current study showed that N-methyl-D-aspartate (NMDA) receptors have a role in spatial learning and memory, indicating that administration of D-AP5 (NMDA receptor antagonist) induced spatial memory impairment, while administration of NMDA facilitated spatial learning (Parsaei et al., 2016[60]). Chronic lithium treatment increases glutamate synaptosomal uptake and decreases hippocampal GluR1 expression in the hippocampus, which has beneficial effects on treatment of mood disorders (Du et al., 2004[16]; Hashimoto et al., 2002[25]). Also, it showed that lithium enhances VGLUT1 (necessary for glutamate uptake) mRNA expression in the cerebral cortex neurons (Moutsimilli et al., 2005[53]). These effects of lithium on the balance of glutamate and GSK3-β activity may lead to the impairment of spatial memory retrieval. Specifics of our experimental findings are outlined below.

### Spatial memory impairment effect consequence of ACPA, but not AM251 administration 

Cannabinoid drugs, ACPA (CB1r agonist) and AM251 (CB1r antagonist), increased escape latency and traveled distance to the platform, suggesting a spatial learning impairment (Figure 1A, B(Fig. 1), Figure 2A, B(Fig. 2), respectively). In the probe test, ACPA impaired spatial memory retrieval, while AM251 did not impair (Figure 4(Fig. 4)). Also, both ACPA and AM251 decreased swimming speed, however, increased swimming speed in one trial (trial 6) (Figure 1C(Fig. 1) and Figure 2C(Fig. 2), respectively). Previous studies showed that CB1r agonists, such as ACPA, induce cognitive impairments in rodents (Kruk-Slomka and Biala, 2016[36]; Kruk-Slomka et al., 2016[37]; Pamplona and Takahashi, 2006[58]), But CB1r antagonists, such as AM251, often enhance the performance of rodents in different memory tasks (Kruk-Slomka and Biala, 2016[36]; Lichtman, 2000[41]; Takahashi et al., 2005[76]). The effect of cannabinoids on locomotor activity is inconsistent. In some researches, cannabinoids increased locomotor activity and in some researches decreased it (Chaperon and Thiebot, 1999[7]; Sulcova et al., 1998[74]). Acute administration of Δ9-tetrahydrocannabinol (Δ9-THC) or CB1r agonist impairs acquisition of memory in rodents (Da and Takahashi, 2002[11]; Lichtman et al., 1995[42]). Cannabinoids reduce glutamatergic synaptic transmission in different brain regions which have important roles in memory-related functions (Azad et al., 2003[4]; Fujiwara and Egashira, 2004[21]; Robbe et al., 2001[67]). Generally, activation of CB1r in the hippocampus, inhibits synaptic transmission (Hoffman and Lupica, 2000[26]). CB1r activation reduces LTP and inhibits the release of glutamate in the hippocampus (Sullivan, 2000[75]). Cannabinoids have influence on the function of NMDA receptors via various ways, such as reduction of releasing presynaptic glutamate into the synaptic cleft (Li et al., 2011[39]). However, it has been described that AM251 and other CB1r antagonists increased extracellular glutamate (Xi et al., 2006[82]). Cannabinoids have modulation effect on hippocampal glutamatergic activation, because releasing of intracellular calcium which have mediated by NMDA receptors, reduced by CB1r activation (Hampson et al., 2011[24]). CB1r activation reduces synaptic processing in the hippocampus, and also, reduces flexibility and adaptation of neurons for successful memory encoding and retrieval by decreasing the modulation of intracellular calcium releasing in critical circumstances (Deadwyler et al., 2007[12]; Deadwyler and Hampson, 2008[13]). NMDA receptors are essential for brain development, synaptic plasticity and direct effect on learning and memory consolidation (Dingledine et al., 1999[15]). However, it is notable that the reducing effect of cannabinoids on glutamate releasing may occur by other mechanisms unrelated to the CB1r (Sanchez-Blazquez et al., 2014[68]). Also, cannabinoids such as THC, inactivate GSK3-β. CB1r mediates this effect, because blocking this receptor by CB1r antagonists abolish the phosphorylation of GSK3-β (Ozaita et al., 2007[57]). Spatial memory impairment induced by cannabinoids may occur for their reducing effect on glutamate releasing, and also, for their inhibiting effect on GSK3-β.

### The similar dual effect of both cannabinoid drugs on lithium-induced spatial memory impairment

Following the existing literature, we designed some experiments to elucidate the consequences of interaction between lithium and cannabinoids on spatial memory in MWM apparatus. The results showed that in the trial session, administration of lower dose of ACPA (0.001 µg/rat) after injection of lithium (0.5 mg/kg) increased both escape latency and traveled distance during spatial learning (trial 3). Also, administration of lower dose of AM251 (1 ng/rat) after injection of lithium (1 mg/kg) increased only escape latency during spatial learning (trial 6) (Figure 3A, B(Fig. 3)). In the probe test, both lower doses of cannabinoid drugs restored the spatial memory impairment effect of the higher dose of lithium (5 mg/kg), while potentiated the spatial memory impairment effect of lower doses of lithium (0.5 and 1 mg/kg) (Figure 5(Fig. 5)). Interaction between ACPA and lithium (5 mg/kg) increased swimming speed only in trial 3, and interaction between AM251 and lithium (1 mg/kg) increased swimming speed only in trial 8 (Figure 3C(Fig. 3)). As a result, the data showed that lower doses of ACPA (0.001 µg/rat) and AM251 (1 ng/rat), have dual effect on lithium-induced spatial memory impairment in rats. Previous reports demonstrated that cannabinoids may have dual effects in different experiments (Li et al., 2013[40]; Sarne et al., 2011[69]). For example, some experiments showed that cannabinoids decrease body temperature, inhibit nociception and suppress motor activity (Ameri, 1999[2]; Chaperon and Thiebot, 1999[7]). But nevertheless, many experiments showed opposite results: hyperalgesia, increasing motor activity and elevating body temperature (Sulcova et al., 1998[74]; Taylor and Fennessy, 1977[77]). Previous studies showed that acute administration of cannabinoids has protective effects in different models of acute brain injuries. However, chronic activation of CB receptors has neurotoxic consequences in heavy cannabis users (Arnone et al., 2008[3]; Ehrenreich et al., 1999[18]; Matochik et al., 2005[50]; McHale and Hunt, 2008[51]; Solowij et al., 2002[71]). Also, previous study showed that the CB1r agonist, ACPA, has dual effect on memory in step-down passive avoidance learning task (Nasehi et al., 2015[55]). CB1r peptide agonists RVD and VD, impair memory processing in mice, but in Amyloid-beta_1_-_42_-treated mice, RVD and VD improve Aβ_1-42_-induced memory impairment (Zhang et al., 2016[83]). Another research showed that Cannabidiol (CBD) has dual effect on lithium-induced vomiting, indicating that lower doses of CBD suppressed lithium-induced vomiting, while higher doses enhanced it (Parker et al., 2004[59]). In this research, both cannabinoid drugs, ACPA and AM251, had similar effect on lithium-induced spatial memory impairment. The similar effect of CB receptor agonists and antagonists has been revealed in some experiments before. For instance, in the test of unconditioned fear, both selective CB1r agonist, ACEA, and selective CB1r antagonist, AM251, increased anxiety-like behavior (Simone et al., 2015[70]). Micromolar concentrations of WIN55212-2 (non-selective CB1r agonist), WIN55212-3 (selective CB2r antagonist) and AM251 (selective CB1r antagonist) inhibit the uptake of dopamine in striatal synaptosomes. Also, both WIN55212-2 and AM251, displace the binding of the cocaine analogue, [3H]WIN35,428, indicating a direct interaction with the transporter (Price et al., 2007[65]). In another research, results showed that AM251 failed to reverse WIN-induced inhibition of dopamine uptake in neocortical synaptosomes in rats (Steffens and Feuerstein, 2004[72]). In a related study, results showed that SR141716A (Rimonabant), the CB1r antagonist, has an inhibitory effect on forskolin-stimulated cyclic AMP accumulation. This effect is similar to the effect of cannabinoid agonists, suggests it may be acting as a low intrinsic activity agonist, rather than a pure antagonist at CB1r (Holland et al., 1999[27]). 

## Conclusion

Previous studies revealed important interactions for lithium and cannabinoids. We know that lithium and cannabinoids inhibit GSK3-β and modulate ERK1/2, and cannabinoids may present dual effects in various experimental tasks. Also, CBr agonists and antagonists may have similar effects in different experiments. About the dual effect of cannabinoids, we have two hypotheses. First, the dual effect of CB1r agonist and antagonist (ACPA and AM251) on lithium-induced spatial memory impairment may have related to the dual effect of cannabinoids on lithium-induced GSK3-β modulation. Second, cannabinoids may present a dual effect in lithium-induced glutamate releasing changes and may effect on glutamate releasing pattern in the CA1 hippocampal region. As we said before, NMDA glutamate receptors have a critical role in spatial memory. On the other hand, for the similar effect of cannabinoids we have three hypotheses, too. First, the same effect of CB1r agonist and antagonist (ACPA and AM251) may have related to the modality of AM251's function. As we said above, low agonist activity of CB1r antagonists such as AM251 and SR141716A had been shown in some cognitive tasks. The reason of agonist-like effects of CB1r antagonists may be related to the diversity of types or subtypes of cannabinoid receptors that are distinct from the CB1r. Also, it may occur when the agonist-like effect of CB1r antagonist is predominated over its antagonist effect in some specific tasks. Second, effects of cannabinoid drugs may be dose-dependent. It has been revealed that cannabinoids in different doses and also, in interfering with different doses of other drugs, have shown different effects. Third, the sensitivity of CB1r to cannabinoid drugs may variable in different regions of the brain, because the effect of cannabinoids is different across various tasks.

In conclusion, cannabinoids may have a dual effect on lithium-induced spatial memory impairment in rats, and this effect may consequence of probable dual effect of cannabinoids on lithium-induced GSK3-β functional changes, and their interaction effect with lithium on NMDA glutamate receptor's activity in the CA1 region.

## Supplementary Material

Supplementary data
