# Supplementary material for: The role of CA1 CB1 receptors on lithium-induced spatial memory impairment in rats
Source: EXCLI J. 2018 Sep 20;17:916–34. doi: 10.17179/excli2018-1511 (PMC6295625; doi:10.17179/excli2018-1511)
Supplement: Supplementary data [file EXCLI-17-916-s-001.pdf]

EXCLI Journal 2018;17:916-934 – ISSN 1611-2156
Received: July 07, 2018, accepted: August 27, 2018, published: September 20, 2018

Supplementary data to:

THE ROLE OF CA1 CB1 RECEPTORS ON LITHIUM-INDUCED SPATIAL MEMORY IMPAIRMENT IN RATS

Salar Vaseghi[1], Vahab Babapour[2], Mohammad Nasehi[3*], Mohammad-Reza Zarrindast[4,5,6]

[1] Department of Physiology, Faculty of Veterinary Science, Science and Research Branch, Islamic Azad University, Tehran, Iran
[2] Department of Basic Sciences, Faculty of Veterinary Medicine, University of Tehran, Tehran, Iran
[3] Cognitive and Neuroscience Research Center (CNRC), Amir-Almomenin Hospital, Tehran Medical Sciences Branch, Islamic Azad University, Tehran, Iran
[4] Department of Pharmacology School of Medicine, Tehran University of Medical Sciences, Tehran, Iran
[5] Institute for Cognitive Science Studies (ICSS), Tehran, Iran
[6] Department of Neuroendocrinology, Endocrinology and Metabolism Research Institute, Tehran University of Medical Sciences, Tehran, Iran

* Corresponding author: M. Nasehi (Nasehi@iricss.org; Cognitive and Neuroscience Research Center (CNRC), Amir-Almomenin Hospital, Tehran Medical Sciences Branch, Islamic Azad University, Tehran, Iran). P.O. Box 13145-784, Tel: +9821-99881118-20, Fax: +9821-99881117

http://dx.doi.org/10.17179/excli2018-1511

This is an Open Access article distributed under the terms of the Creative Commons Attribution License (http://creativecommons.org/licenses/by/4.0/).

EXCLI Journal 2018;17:916-934 – ISSN 1611-2156
Received: July 07, 2018, accepted: August 27, 2018, published: September 20, 2018

Raw Data

Escape latency (Time): sec
Traveled distance: cm
Swimming speed: cm/sec

Trials

Supplementary Tables “1” for Figure 1:
Effects of ACPA on spatial learning and swimming speed. Four groups of eight animals received pre-training intra-CA1 administration of saline (1 µl/rat) or different doses of ACPA (0.001, 0.01 and 1 µg/rat). 5 min after the injection animals were trained in MWM apparatus.

Figure 1A: Effects of ACPA on escape latency (Time)

Rat	Trial 1	Trial 2	Trial 3	Trial 4	Trial 5	Trial 6	Trial 7	Trial 8
**1**	49	39	27	22	16	12	7	6
**2**	46	35	29	17	14	11	7	7
**3**	44	36	26	19	14	10	6	6
**4**	47	40	30	21	19	12	8	8
**5**	60	47	31	24	18	14	7	9
**6**	50	42	29	19	15	10	9	7
**7**	60	49	33	25	17	12	7	6
**8**	52	41	27	22	18	11	8	9

Figure 1A: Effects of ACPA on escape latency (Time): Saline

Rat	Trial 1	Trial 2	Trial 3	Trial 4	Trial 5	Trial 6	Trial 7	Trial 8
**1**	60	53	44	32	29	27	22	19
**2**	51	42	40	33	31	26	20	22
**3**	60	46	38	32	28	22	22	19
**4**	56	60	41	36	29	25	22	18
**5**	47	39	33	25	26	28	21	18
**6**	42	38	37	33	30	27	25	20
**7**	51	43	40	30	28	25	27	19
**8**	60	60	40	32	26	29	25	17

Figure 1A: Effects of ACPA on escape latency (Time): ACPA 0.001 µg/rat

Rat	Trial 1	Trial 2	Trial 3	Trial 4	Trial 5	Trial 6	Trial 7	Trial 8
**1**	60	52	46	35	37	27	24	21
**2**	56	60	42	40	36	33	22	26
**3**	60	51	40	43	33	35	31	28
**4**	60	60	53	48	43	33	29	25
**5**	47	40	32	26	27	22	18	19
**6**	42	60	50	36	31	22	28	24
**7**	50	42	35	30	29	24	19	20
**8**	60	49	45	39	33	26	23	23

Figure 1A: Effects of ACPA on escape latency (Time): ACPA 0.01 µg/rat

Rat	Trial 1	Trial 2	Trial 3	Trial 4	Trial 5	Trial 6	Trial 7	Trial 8
**1**	60	52	46	35	32	24	22	24
**2**	60	49	39	30	29	30	26	25
**3**	60	60	41	40	35	33	26	29
**4**	55	60	37	40	35	27	24	26
**5**	45	52	38	29	40	21	20	18
**6**	47	43	41	36	33	27	24	21
**7**	52	60	51	41	36	31	29	25
**8**	60	60	48	38	39	33	24	26

Figure 1A: Effects of ACPA on escape latency (Time): ACPA 1 µg/rat

EXCLI Journal 2018;17:916-934 – ISSN 1611-2156
Received: July 07, 2018, accepted: August 27, 2018, published: September 20, 2018

Figure 1B: Effects of ACPA on traveled distance

Rat	Trial 1	Trial 2	Trial 3	Trial 4	Trial 5	Trial 6	Trial 7	Trial 8
1	1467	1210	891	756	535	387	232	222
2	1398	1110	943	589	422	349	250	241
3	1366	1154	852	652	431	316	212	209
4	1424	1245	989	737	666	366	287	291
5	1767	1412	1042	792	623	404	230	287
6	1571	1291	958	670	452	296	302	245
7	1788	1491	1123	842	587	376	240	223
8	1612	1287	876	755	617	345	292	310

Figure 1B: Effects of ACPA on traveled distance: Saline

Rat	Trial 1	Trial 2	Trial 3	Trial 4	Trial 5	Trial 6	Trial 7	Trial 8
1	1744	1622	1346	1067	930	875	729	666
2	1591	1326	1261	1081	1008	845	701	724
3	1771	1404	1167	1051	879	699	731	678
4	1690	1786	1255	1131	919	804	702	621
5	1414	1225	1089	825	845	896	691	634
6	1311	1187	1125	1090	955	866	798	689
7	1579	1341	1255	977	888	810	845	633
8	1759	1764	1241	1025	843	900	808	591

Figure 1B: Effects of ACPA on traveled distance: ACPA 0.001 µg/rat

Rat	Trial 1	Trial 2	Trial 3	Trial 4	Trial 5	Trial 6	Trial 7	Trial 8
1	1755	1588	1378	1091	1100	874	790	732
2	1677	1791	1333	1269	1102	1043	701	878
3	1773	1511	1246	1378	1003	1123	994	915
4	1821	1794	1619	1498	1389	1089	946	818
5	1447	1245	988	822	829	732	612	698
6	1331	1811	1525	1200	979	712	911	803
7	1566	1347	1145	989	941	789	655	707
8	1721	1498	1409	1241	1002	828	738	776

Figure 1B: Effects of ACPA on traveled distance: ACPA 0.01 µg/rat

Rat	Trial 1	Trial 2	Trial 3	Trial 4	Trial 5	Trial 6	Trial 7	Trial 8
1	1744	1618	1411	1111	1077	833	746	795
2	1812	1522	1158	978	941	965	865	805
3	1789	1750	1278	1288	1109	1100	808	951
4	1671	1733	1155	1259	1135	904	798	869
5	1393	1605	1201	944	1300	733	725	635
6	1423	1406	1324	1189	1005	861	778	745
7	1599	1772	1597	1265	1120	1001	977	810
8	1753	1824	1510	1178	1172	1084	800	867

Figure 1B: Effects of ACPA on traveled distance: ACPA 1 µg/rat

EXCLI Journal 2018;17:916-934 – ISSN 1611-2156
Received: July 07, 2018, accepted: August 27, 2018, published: September 20, 2018

Figure 1C: Effects of ACPA on swimming speed

Rat	Trial 1	Trial 2	Trial 3	Trial 4	Trial 5	Trial 6	Trial 7	Trial 8
1	29.93	31.02	33.00	34.36	33.43	32.25	33.14	37.00
2	30.39	31.71	32.51	34.64	30.14	31.72	35.71	34.42
3	31.04	32.05	32.76	34.31	30.78	31.60	35.33	34.83
4	30.29	31.12	32.96	35.09	35.05	30.50	35.87	36.37
5	29.45	30.04	33.61	33.00	34.61	28.85	32.85	31.88
6	31.42	30.73	33.03	35.26	30.13	29.60	33.55	35.00
7	29.80	30.42	34.03	33.68	34.52	31.33	34.28	37.16
8	31.00	31.39	32.44	34.31	34.27	31.36	36.50	34.44

Figure 1C: Effects of ACPA on swimming speed: Saline

Rat	Trial 1	Trial 2	Trial 3	Trial 4	Trial 5	Trial 6	Trial 7	Trial 8
1	29.06	30.60	30.59	33.34	32.06	32.40	33.13	35.05
2	31.19	31.57	31.52	32.75	32.51	32.50	35.05	32.90
3	29.51	30.52	30.71	32.84	31.39	31.77	33.22	35.68
4	30.17	29.76	30.60	31.41	31.68	32.16	31.90	34.50
5	30.08	31.41	33.00	33.00	32.50	32.00	32.90	35.22
6	31.21	31.22	30.40	33.03	31.83	32.07	31.92	34.45
7	30.96	31.18	31.37	32.56	31.71	32.40	31.29	33.31
8	29.31	29.40	31.02	32.03	32.42	31.03	32.32	34.76

Fiure 1C: Effects of ACPA on swimming speed: ACPA 0.001 µg/rat

Rat	Trial 1	Trial 2	Trial 3	Trial 4	Trial 5	Trial 6	Trial 7	Trial 8
1	29.25	30.53	29.95	31.17	29.72	32.37	32.91	34.85
2	29.94	29.85	31.73	31.72	30.61	31.60	31.86	33.76
3	29.55	29.62	31.15	32.04	30.39	32.08	32.06	32.67
4	30.35	29.90	30.54	31.20	32.30	33.00	32.62	32.72
5	30.78	31.12	30.87	31.61	30.70	33.27	34.00	36.73
6	31.69	30.18	30.50	33.33	31.58	32.36	32.53	33.45
7	31.32	32.07	32.71	32.96	32.44	32.87	34.47	35.35
8	28.68	30.57	31.31	31.82	30.36	31.84	32.08	33.73

Figure 1C: Effects of ACPA on swimming speed: ACPA 0.01 µg/rat

Rat	Trial 1	Trial 2	Trial 3	Trial 4	Trial 5	Trial 6	Trial 7	Trial 8
1	29.06	31.11	30.67	31.74	33.65	34.70	33.90	33.12
2	30.20	31.06	29.69	32.60	32.44	32.16	33.26	32.20
3	29.81	29.16	31.17	32.20	31.68	33.33	31.07	32.79
4	30.38	28.88	31.21	31.47	32.42	33.48	33.25	33.42
5	30.95	30.86	31.60	32.55	32.50	34.90	36.25	35.27
6	30.27	32.69	32.29	33.02	30.45	31.88	32.41	35.47
7	30.75	29.53	31.31	30.85	31.11	32.29	33.68	32.40
8	29.21	30.40	31.45	31.00	30.05	32.84	33.33	33.34

Figure 1C: Effects of ACPA on swimming speed: ACPA 1 µg/rat

EXCLI Journal 2018;17:916-934 – ISSN 1611-2156
Received: July 07, 2018, accepted: August 27, 2018, published: September 20, 2018

Raw Data

Escape latency (Time): sec
Traveled distance: cm
Swimming speed: cm/sec

Trials

Supplementary Tables "2" for Figure 2:
Effects of AM251 on spatial learning and swimming speed. Four groups of eight animals received pre-training intra-CA1 administration of saline (1 µl/rat) or different doses of AM251 (1, 10 and 100 ng/rat). 5 min after the injection animals were trained in MWM apparatus.

EXCLI Journal 2018;17:916-934 – ISSN 1611-2156
Received: July 07, 2018, accepted: August 27, 2018, published: September 20, 2018

Figure 2A: Effects of AM251 on escape latency (Time)

Rat	Trial 1	Trial 2	Trial 3	Trial 4	Trial 5	Trial 6	Trial 7	Trial 8
1	49	39	27	22	16	12	7	6
2	46	35	29	17	14	11	7	7
3	44	36	26	19	14	10	6	6
4	47	40	30	21	19	12	8	8
5	60	47	31	24	18	14	7	9
6	50	42	29	19	15	10	9	7
7	60	49	33	25	17	12	7	6
8	52	41	27	22	18	11	8	9

Figure 2A: Effects of AM251 on escape latency (Time): Saline

Rat	Trial 1	Trial 2	Trial 3	Trial 4	Trial 5	Trial 6	Trial 7	Trial 8
1	49	53	39	33	30	26	18	17
2	60	52	38	35	26	22	21	20
3	52	51	42	31	24	29	22	21
4	52	37	32	25	26	28	24	18
5	60	42	35	32	32	24	19	17
6	53	44	39	31	29	22	24	18
7	60	41	40	27	29	29	20	15
8	52	45	41	36	32	24	25	19

Figure 2A: Effects of AM251 on escape latency (Time): AM251 1 ng/rat

Rat	Trial 1	Trial 2	Trial 3	Trial 4	Trial 5	Trial 6	Trial 7	Trial 8
1	60	53	42	32	29	25	26	20
2	53	60	52	35	19	22	23	15
3	60	53	45	29	22	24	18	14
4	60	46	42	25	27	19	21	22
5	45	43	38	30	32	28	27	18
6	55	45	42	39	34	25	19	20
7	60	53	42	37	29	26	22	16
8	51	46	37	41	31	25	29	20

Figure 2A: Effects of AM251 on escape latency (Time): AM251 10 ng/rat

Rat	Trial 1	Trial 2	Trial 3	Trial 4	Trial 5	Trial 6	Trial 7	Trial 8
1	60	43	44	21	22	18	22	19
2	58	51	43	27	29	25	23	21
3	51	45	35	38	35	23	25	16
4	60	60	45	35	30	20	23	15
5	60	58	44	35	23	23	20	19
6	58	44	47	42	29	21	19	20
7	48	49	34	37	24	30	25	21
8	51	60	47	36	40	26	29	18

Figure 2A: Effects of AM251 on escape latency (Time): AM251 100 ng/rat

EXCLI Journal 2018;17:916-934 – ISSN 1611-2156
Received: July 07, 2018, accepted: August 27, 2018, published: September 20, 2018

Figure 2B: Effects of AM251 on traveled distance

Rat	Trial 1	Trial 2	Trial 3	Trial 4	Trial 5	Trial 6	Trial 7	Trial 8
1	1467	1210	891	756	535	387	232	222
2	1398	1110	943	589	422	349	250	241
3	1366	1154	852	652	431	316	212	209
4	1424	1245	989	737	666	366	287	291
5	1767	1412	1042	792	623	404	230	287
6	1571	1291	958	670	452	296	302	245
7	1788	1491	1123	842	587	376	240	223
8	1612	1287	876	755	617	345	292	310

Figure 2B: Effects of AM251 on traveled distance: Saline

Rat	Trial 1	Trial 2	Trial 3	Trial 4	Trial 5	Trial 6	Trial 7	Trial 8
1	1433	1645	1214	1091	969	850	590	584
2	1741	1605	1184	1144	861	712	694	670
3	1600	1567	1311	978	790	933	731	702
4	1589	1154	1084	821	856	893	761	603
5	1756	1290	1092	1010	1041	778	648	562
6	1626	1354	1178	966	926	742	795	584
7	1760	1245	1234	900	911	949	681	438
8	1617	1337	1228	1187	1014	782	784	610

Figure 2B: Effects of AM251 on traveled distance: AM251 1 ng/rat

Rat	Trial 1	Trial 2	Trial 3	Trial 4	Trial 5	Trial 6	Trial 7	Trial 8
1	1757	1674	1265	1032	943	768	806	700
2	1598	1795	1632	1110	621	704	745	422
3	1734	1654	1322	922	723	758	567	387
4	1765	1362	1241	823	878	643	698	745
5	1310	1256	1146	974	1035	916	871	590
6	1622	1302	1255	1235	1099	777	620	679
7	1751	1641	1240	1189	926	789	732	541
8	1590	1345	1206	1214	981	762	951	688

Figure 2B: Effects of AM251 on traveled distance: AM251 10 ng/rat

Rat	Trial 1	Trial 2	Trial 3	Trial 4	Trial 5	Trial 6	Trial 7	Trial 8
1	1768	1278	1287	694	722	589	734	624
2	1761	1482	1284	809	879	814	758	685
3	1600	1332	1125	1220	1178	774	800	521
4	1805	1745	1325	1119	1001	701	734	487
5	1758	1702	1293	1141	750	742	671	642
6	1721	1298	1387	1254	941	690	612	685
7	1398	1444	1108	1178	731	967	820	720
8	1489	1764	1360	1125	1170	784	960	595

Figure 2B: Effects of AM251 on traveled distance: AM251 100 ng/rat

EXCLI Journal 2018;17:916-934 – ISSN 1611-2156
Received: July 07, 2018, accepted: August 27, 2018, published: September 20, 2018

Figure 2C: Effects of AM251 on swimming speed

Rat	Trial 1	Trial 2	Trial 3	Trial 4	Trial 5	Trial 6	Trial 7	Trial 8
1	29.93	31.02	33.00	34.36	33.43	32.25	33.14	37.00
2	30.39	31.71	32.51	34.64	30.14	31.72	35.71	34.42
3	31.04	32.05	32.76	34.31	30.78	31.60	35.33	34.83
4	30.29	31.12	32.96	35.09	35.05	30.50	35.87	36.37
5	29.45	30.04	33.61	33.00	34.61	28.85	32.85	31.88
6	31.42	30.73	33.03	35.26	30.13	29.60	33.55	35.00
7	29.80	30.42	34.03	33.68	34.52	31.33	34.28	37.16
8	31.00	31.39	32.44	34.31	34.27	31.36	36.50	34.44

Figure 2C: Effects of AM251 on swimming speed: Saline

Rat	Trial 1	Trial 2	Trial 3	Trial 4	Trial 5	Trial 6	Trial 7	Trial 8
1	29.24	31.03	31.12	33.06	32.30	32.69	32.77	34.35
2	29.01	30.86	31.15	32.68	33.11	32.36	33.04	33.50
3	30.76	30.72	31.21	31.54	32.91	32.17	33.22	33.42
4	30.55	31.18	33.87	32.84	32.92	31.89	31.70	33.50
5	29.26	30.71	31.20	31.56	32.53	32.41	34.10	33.05
6	30.67	30.77	30.20	31.16	31.93	33.72	33.12	32.44
7	29.33	30.36	30.85	33.33	31.41	32.72	34.05	29.20
8	31.09	29.71	29.95	32.97	31.68	32.58	31.36	32.10

Figure 2C: Effects of AM251 on swimming speed: AM251 1 ng/rat

Rat	Trial 1	Trial 2	Trial 3	Trial 4	Trial 5	Trial 6	Trial 7	Trial 8
1	29.28	31.58	30.11	32.25	32.51	30.72	31.00	35.00
2	30.15	29.91	31.38	31.71	32.68	32.00	32.32	28.13
3	28.90	31.20	29.37	31.79	32.86	31.58	31.50	27.64
4	29.41	29.60	29.54	32.92	32.51	33.84	33.23	33.86
5	29.11	29.20	30.15	32.46	32.34	32.71	32.25	32.77
6	29.42	28.93	29.88	31.66	32.32	31.08	32.63	33.95
7	29.18	30.96	29.52	32.13	31.93	30.34	33.27	33.81
8	31.17	29.23	32.59	29.60	31.64	30.48	32.79	34.40

Figure 2C: Effects of AM251 on swimming speed: AM251 10 ng/rat

Rat	Trial 1	Trial 2	Trial 3	Trial 4	Trial 5	Trial 6	Trial 7	Trial 8
1	29.46	29.72	29.25	33.04	32.81	32.72	33.36	32.84
2	30.36	29.05	29.86	29.96	30.38	32.56	32.95	32.61
3	31.37	29.60	32.14	32.10	33.65	33.65	32.00	32.56
4	30.08	29.08	29.44	31.97	33.36	35.05	31.91	32.46
5	29.30	29.34	29.38	32.60	32.60	32.26	33.55	33.78
6	29.67	29.50	29.51	29.85	32.44	32.85	32.21	34.25
7	29.12	29.46	32.58	31.83	30.45	32.23	32.80	34.28
8	29.19	29.40	28.93	31.25	29.25	30.15	33.10	33.05

Figure 2C: Effects of AM251 on swimming speed: AM251 100 ng/rat

Received: July 07, 2018, accepted: August 27, 2018, published: September 20, 2018

Raw Data

Escape latency (Time): sec
Traveled distance: cm
Swimming speed: cm/sec

Trials

Supplementary Tables “3” for Figure 3:
Effects of lithium, and interaction between cannabinoids and lithium on spatial learning and swimming speed. The animals received pre-training intraperitoneal administration of saline (1 ml/kg) or different doses of lithium (0.5, 1 and 5 mg/kg). 5 min after pervious injection the animals received lower dose of ACPA (0.001 µg/rat; four groups, eight rats in each group) or AM251 (1 ng/rat; four groups, eight rats in each group).

EXCLI Journal 2018;17:916-934 – ISSN 1611-2156
Received: July 07, 2018, accepted: August 27, 2018, published: September 20, 2018

Figure 3A: Effects of Lithium on escape latency (Time)

Rat	Trial 1	Trial 2	Trial 3	Trial 4	Trial 5	Trial 6	Trial 7	Trial 8
**1**	49	39	27	22	16	12	7	6
**2**	46	35	29	17	14	11	7	7
**3**	44	36	26	19	14	10	6	6
**4**	47	40	30	21	19	12	8	8
**5**	60	47	31	24	18	14	7	9
**6**	50	42	29	19	15	10	9	7
**7**	60	49	33	25	17	12	7	6
**8**	52	41	27	22	18	11	8	9

Figure 3A: Effects of Lithium on escape latency (Time): Saline

Rat	Trial 1	Trial 2	Trial 3	Trial 4	Trial 5	Trial 6	Trial 7	Trial 8
**1**	60	48	26	22	17	10	12	6
**2**	60	60	37	28	15	11	7	8
**3**	47	45	24	27	14	14	8	6
**4**	42	25	28	22	15	19	10	10
**5**	55	34	29	18	20	9	10	6
**6**	51	40	21	15	14	9	17	9
**7**	60	31	27	25	19	11	12	10
**8**	44	33	19	24	11	15	7	8

Figure 3A: Effects of Lithium on escape latency (Time): Lithium 0.5 mg/kg

Rat	Trial 1	Trial 2	Trial 3	Trial 4	Trial 5	Trial 6	Trial 7	Trial 8
**1**	60	51	31	27	21	11	6	9
**2**	53	60	27	21	23	14	7	11
**3**	45	34	29	19	23	8	8	6
**4**	60	39	33	20	15	14	12	5
**5**	60	50	27	14	11	16	13	6
**6**	56	37	42	28	22	11	9	11
**7**	41	44	31	22	19	9	14	9
**8**	43	57	35	17	21	14	12	10

Figure 3A: Effects of Lithium on escape latency (Time): Lithium 1 mg/kg

Rat	Trial 1	Trial 2	Trial 3	Trial 4	Trial 5	Trial 6	Trial 7	Trial 8
**1**	55	60	41	22	23	14	9	7
**2**	42	35	26	31	18	15	6	6
**3**	60	36	40	28	19	14	7	8
**4**	60	51	33	21	15	20	13	7
**5**	60	44	39	25	22	11	12	11
**6**	39	28	33	18	21	12	10	10
**7**	55	41	36	19	15	8	11	8
**8**	42	29	30	24	24	15	11	8

Figure 3A: Effects of Lithium on escape latency (Time): Lithium 5 mg/kg

EXCLI Journal 2018;17:916-934 – ISSN 1611-2156
Received: July 07, 2018, accepted: August 27, 2018, published: September 20, 2018

Figure 3A: Effects of ACPA 0.001 µg/rat + Lithium on escape latency (Time)

Rat	Trial 1	Trial 2	Trial 3	Trial 4	Trial 5	Trial 6	Trial 7	Trial 8
**1**	60	53	44	32	29	27	22	19
**2**	51	42	40	33	31	26	20	22
**3**	60	46	38	32	28	22	22	19
**4**	56	60	41	36	29	25	22	18
**5**	47	39	33	25	26	28	21	18
**6**	42	38	37	33	30	27	25	20
**7**	51	43	40	30	28	25	27	19
**8**	60	60	40	32	26	29	25	17

Figure 3A: Effects of ACPA 0.001 µg/rat + Lithium on escape latency (Time):
ACPA 0.001 µg/rat + Saline

Rat	Trial 1	Trial 2	Trial 3	Trial 4	Trial 5	Trial 6	Trial 7	Trial 8
**1**	52	46	33	24	19	13	7	9
**2**	60	60	40	25	20	16	8	7
**3**	60	44	38	21	20	14	6	8
**4**	60	49	37	27	22	14	10	7
**5**	48	41	31	17	19	16	11	8
**6**	60	44	32	19	13	11	7	11
**7**	42	53	37	22	17	14	10	9
**8**	49	35	29	18	14	11	6	10

Figure 3A: Effects of ACPA 0.001 µg/rat + Lithium on escape latency (Time):
ACPA 0.001 µg/rat + Lithium 0.5 mg/kg

Rat	Trial 1	Trial 2	Trial 3	Trial 4	Trial 5	Trial 6	Trial 7	Trial 8
**1**	60	48	33	21	19	15	10	6
**2**	60	42	27	25	17	14	9	7
**3**	48	53	29	21	18	16	9	6
**4**	41	32	36	22	23	22	13	6
**5**	60	48	31	18	16	16	10	9
**6**	55	41	35	19	17	14	7	10
**7**	50	60	41	27	22	19	13	11
**8**	60	60	47	33	21	12	8	7

Figure 3A: Effects of ACPA 0.001 µg/rat + Lithium on escape latency (Time):
ACPA 0.001 µg/rat + Lithium 1 mg/kg

Rat	Trial 1	Trial 2	Trial 3	Trial 4	Trial 5	Trial 6	Trial 7	Trial 8
**1**	60	41	35	21	20	15	10	11
**2**	60	46	32	22	17	18	11	8
**3**	46	36	35	31	22	20	15	11
**4**	41	51	31	24	16	18	11	10
**5**	44	52	39	32	23	16	9	9
**6**	60	41	27	21	16	18	8	7
**7**	58	45	30	24	17	16	9	6
**8**	51	44	37	26	14	13	10	7

Figure 3A: Effects of ACPA 0.001 µg/rat + Lithium on escape latency (Time):
ACPA 0.001 µg/rat + Lithium 5 mg/kg

EXCLI Journal 2018;17:916-934 – ISSN 1611-2156
Received: July 07, 2018, accepted: August 27, 2018, published: September 20, 2018

Figure 3A: Effects of AM251 1 ng/rat + Lithium on escape latency (Time)

Rat	Trial 1	Trial 2	Trial 3	Trial 4	Trial 5	Trial 6	Trial 7	Trial 8
**1**	49	53	39	33	30	26	18	17
**2**	60	52	38	35	26	22	21	20
**3**	52	51	42	31	24	29	22	21
**4**	52	37	32	25	26	28	24	18
**5**	60	42	35	32	32	24	19	17
**6**	53	44	39	31	29	22	24	18
**7**	60	41	40	27	29	29	20	15
**8**	52	45	41	36	32	24	25	19

Figure 3A: Effects of AM251 1 ng/rat + Lithium on escape latency (Time):
AM251 1 ng/rat + Saline

Rat	Trial 1	Trial 2	Trial 3	Trial 4	Trial 5	Trial 6	Trial 7	Trial 8
**1**	60	51	33	20	16	12	8	6
**2**	60	36	35	21	15	10	9	11
**3**	43	34	26	21	12	11	8	10
**4**	54	53	33	22	19	16	11	7
**5**	45	56	41	27	22	16	12	9
**6**	55	45	28	23	15	14	9	6
**7**	60	44	28	33	20	12	12	6
**8**	58	60	48	32	26	14	11	7

Figure 3A: Effects of AM251 1 ng/rat + Lithium on escape latency (Time):
AM251 1 ng/rat + Lithium 0.5 mg/kg

Rat	Trial 1	Trial 2	Trial 3	Trial 4	Trial 5	Trial 6	Trial 7	Trial 8
**1**	52	43	33	19	24	20	12	8
**2**	52	60	41	22	17	14	10	7
**3**	60	46	31	17	15	15	9	8
**4**	58	43	32	22	24	20	16	11
**5**	60	55	40	32	26	18	10	7
**6**	48	36	31	26	21	15	7	8
**7**	51	41	27	21	22	17	9	6
**8**	42	44	29	24	18	14	11	6

Figure 3A: Effects of AM251 1 ng/rat + Lithium on escape latency (Time):
AM251 1 ng/rat + Lithium 1 mg/kg

Rat	Trial 1	Trial 2	Trial 3	Trial 4	Trial 5	Trial 6	Trial 7	Trial 8
**1**	53	36	34	19	17	11	10	7
**2**	50	32	38	26	17	19	12	11
**3**	60	42	29	22	21	17	11	9
**4**	60	51	35	34	22	19	14	11
**5**	60	39	29	21	17	12	8	8
**6**	60	60	47	27	19	13	9	8
**7**	46	44	33	28	21	19	15	7
**8**	49	41	37	22	15	14	10	7

Figure 3A: Effects of AM251 1 ng/rat + Lithium on escape latency (Time):
AM251 1 ng/rat + Lithium 5 mg/kg

Figure 3B: Effects of Lithium on traveled distance

Rat	Trial 1	Trial 2	Trial 3	Trial 4	Trial 5	Trial 6	Trial 7	Trial 8
1	1467	1210	891	756	535	387	232	222
2	1398	1110	943	589	422	349	250	241
3	1366	1154	852	652	431	316	212	209
4	1424	1245	989	737	666	366	287	291
5	1767	1412	1042	792	623	404	230	287
6	1571	1291	958	670	452	296	302	245
7	1788	1491	1123	842	587	376	240	223
8	1612	1287	876	755	617	345	292	310

Figure 3B: Effects of Lithium on traveled distance: Saline

Rat	Trial 1	Trial 2	Trial 3	Trial 4	Trial 5	Trial 6	Trial 7	Trial 8
1	1745	1444	854	764	575	316	363	187
2	1811	1761	1177	909	500	333	225	266
3	1431	1346	791	877	456	437	278	201
4	1277	829	930	741	494	647	337	324
5	1662	1134	929	578	679	309	341	178
6	1591	1226	711	502	477	295	552	284
7	1770	956	889	820	635	341	372	335
8	1345	1145	632	804	338	511	221	269

Figure 3B: Effects of Lithium on traveled distance: Lithium 0.5 mg/kg

Rat	Trial 1	Trial 2	Trial 3	Trial 4	Trial 5	Trial 6	Trial 7	Trial 8
1	1763	1567	965	908	711	335	210	290
2	1633	1803	871	701	789	481	244	351
3	1361	1141	912	633	771	246	279	189
4	1789	1217	1160	669	504	472	345	146
5	1736	1557	893	454	349	543	379	190
6	1661	1168	1287	913	781	329	288	331
7	1237	1290	978	777	650	266	482	281
8	1311	1701	1200	581	736	491	351	310

Figure 3B: Effects of Lithium on traveled distance: Lithium 1 mg/kg

Rat	Trial 1	Trial 2	Trial 3	Trial 4	Trial 5	Trial 6	Trial 7	Trial 8
1	1631	1770	1257	733	770	478	282	215
2	1242	1188	878	967	478	490	167	200
3	1745	1201	1224	890	512	456	198	244
4	1771	1575	1040	689	455	665	389	225
5	1756	1320	1202	811	709	343	355	359
6	1180	906	1021	631	679	356	303	335
7	1642	1210	1143	666	502	255	319	241
8	1244	922	952	783	771	518	341	258

Figure 3B: Effects of Lithium on traveled distance: Lithium 5 mg/kg

Figure 3B: Effects of ACPA 0.001 µg/rat + Lithium on traveled distance

Rat	Trial 1	Trial 2	Trial 3	Trial 4	Trial 5	Trial 6	Trial 7	Trial 8
1	1744	1622	1346	1067	930	875	729	666
2	1591	1326	1261	1081	1008	845	701	724
3	1771	1404	1167	1051	879	699	731	678
4	1690	1786	1255	1131	919	804	702	621
5	1414	1225	1089	825	845	896	691	634
6	1311	1187	1125	1090	955	866	798	689
7	1579	1341	1255	977	888	810	845	633
8	1759	1764	1241	1025	843	900	808	591

Figure 3B: Effects of ACPA 0.001 µg/rat + Lithium on traveled distance:
ACPA 0.001 µg/rat + Saline

Rat	Trial 1	Trial 2	Trial 3	Trial 4	Trial 5	Trial 6	Trial 7	Trial 8
1	1626	1387	1098	809	662	411	234	321
2	1761	1755	1265	838	688	536	252	251
3	1746	1369	1214	743	701	435	211	230
4	1789	1479	1189	898	738	447	351	250
5	1465	1266	1031	572	641	523	367	287
6	1791	1357	1059	650	398	332	245	352
7	1302	1644	1233	749	577	437	310	279
8	1486	1124	967	623	445	346	219	312

Figure 3B: Effects of ACPA 0.001 µg/rat + Lithium on traveled distance:
ACPA 0.001 µg/rat + Lithium 0.5 mg/kg

Rat	Trial 1	Trial 2	Trial 3	Trial 4	Trial 5	Trial 6	Trial 7	Trial 8
1	1761	1439	1087	751	651	487	325	204
2	1754	1313	889	830	562	455	301	232
3	1445	1667	952	711	613	521	293	211
4	1278	1044	1200	742	787	760	402	209
5	1792	1456	1078	622	558	541	323	267
6	1698	1288	1136	654	583	444	242	299
7	1579	1810	1277	903	756	645	409	332
8	1777	1754	1438	1102	721	379	223	240

Figure 3B: Effects of ACPA 0.001 µg/rat + Lithium on traveled distance:
ACPA 0.001 µg/rat + Lithium 1 mg/kg

Rat	Trial 1	Trial 2	Trial 3	Trial 4	Trial 5	Trial 6	Trial 7	Trial 8
1	1812	1283	1134	742	702	474	312	367
2	1782	1203	1065	783	555	563	355	227
3	1389	1189	1144	1034	771	728	476	353
4	1287	1610	1021	810	509	572	367	312
5	1343	1639	1248	1098	798	514	256	278
6	1767	1276	912	759	493	568	235	234
7	1722	1420	1051	794	526	511	270	204
8	1589	1388	1202	887	438	421	326	224

Figure 3B: Effects of ACPA 0.001 µg/rat + Lithium on traveled distance:
ACPA 0.001 µg/rat + Lithium 5 mg/kg

Figure 3B: Effects of AM251 1 ng/rat + Lithium on traveled distance

Rat	Trial 1	Trial 2	Trial 3	Trial 4	Trial 5	Trial 6	Trial 7	Trial 8
1	1433	1645	1214	1091	969	850	590	584
2	1741	1605	1184	1144	861	712	694	670
3	1600	1567	1311	978	790	933	731	702
4	1589	1154	1084	821	856	893	761	603
5	1756	1290	1092	1010	1041	778	648	562
6	1626	1354	1178	966	926	742	795	584
7	1760	1245	1234	900	911	949	681	438
8	1617	1337	1228	1187	1014	782	784	610

Figure 3B: Effects of AM251 1 ng/rat + Lithium on traveled distance:
AM251 1 ng/rat + Saline

Rat	Trial 1	Trial 2	Trial 3	Trial 4	Trial 5	Trial 6	Trial 7	Trial 8
1	1756	1572	1071	702	501	371	267	212
2	1788	1128	1120	733	446	321	300	345
3	1333	1079	870	731	371	345	281	313
4	1658	1622	1064	759	677	481	376	238
5	1399	1690	1276	879	760	469	391	298
6	1672	1402	901	788	443	406	295	234
7	1746	1378	912	1076	712	391	385	211
8	1721	1792	1448	1025	864	421	345	262

Figure 3B: Effects of AM251 1 ng/rat + Lithium on traveled distance:
AM251 1 ng/rat + Lithium 0.5 mg/kg

Rat	Trial 1	Trial 2	Trial 3	Trial 4	Trial 5	Trial 6	Trial 7	Trial 8
1	1602	1315	1035	648	797	701	398	298
2	1621	1798	1289	751	589	421	321	253
3	1763	1398	998	576	457	445	301	279
4	1712	1321	1012	754	802	689	523	365
5	1801	1677	1281	1008	889	594	309	245
6	1443	1119	1002	865	723	453	234	287
7	1578	1287	893	730	744	602	289	225
8	1300	1366	915	776	610	418	345	221

Figure 3B: Effects of AM251 1 ng/rat + Lithium on traveled distance:
AM251 1 ng/rat + Lithium 1 mg/kg

Rat	Trial 1	Trial 2	Trial 3	Trial 4	Trial 5	Trial 6	Trial 7	Trial 8
1	1645	1156	1087	651	571	332	309	248
2	1564	1076	1203	869	598	661	387	368
3	1777	1298	929	739	711	567	353	296
4	1792	1603	1099	1072	748	656	410	354
5	1812	1212	903	742	564	377	278	288
6	1756	1803	1798	909	639	422	281	276
7	1400	1378	1071	920	728	667	462	239
8	1478	1267	1188	746	433	409	311	241

Figure 3B: Effects of AM251 1 ng/rat + Lithium on traveled distance:
AM251 1 ng/rat + Lithium 5 mg/kg

EXCLI Journal 2018;17:916-934 – ISSN 1611-2156
Received: July 07, 2018, accepted: August 27, 2018, published: September 20, 2018

Figure 3C: Effects of Lithium on swimming speed

Rat	Trial 1	Trial 2	Trial 3	Trial 4	Trial 5	Trial 6	Trial 7	Trial 8
1	29.93	31.02	33.00	34.36	33.43	32.25	33.14	37.00
2	30.39	31.71	32.51	34.64	30.14	31.72	35.71	34.42
3	31.04	32.05	32.76	34.31	30.78	31.60	35.33	34.83
4	30.29	31.12	32.96	35.09	35.05	30.50	35.87	36.37
5	29.45	30.04	33.61	33.00	34.61	28.85	32.85	31.88
6	31.42	30.73	33.03	35.26	30.13	29.60	33.55	35.00
7	29.80	30.42	34.03	33.68	34.52	31.33	34.28	37.16
8	31.00	31.39	32.44	34.31	34.27	31.36	36.50	34.44

Figure 3C: Effects of Lithium on swimming speed: Saline

Rat	Trial 1	Trial 2	Trial 3	Trial 4	Trial 5	Trial 6	Trial 7	Trial 8
1	29.08	30.08	32.84	34.72	33.82	31.60	30.25	31.16
2	30.18	29.35	31.81	32.46	33.33	30.27	32.14	33.25
3	30.44	29.91	32.95	32.48	32.57	31.21	34.75	33.50
4	30.40	33.16	33.21	33.68	32.93	34.05	33.70	32.40
5	30.21	33.35	32.03	32.11	33.95	34.33	34.10	29.66
6	31.19	30.65	33.85	33.46	34.07	32.77	32.47	31.55
7	29.50	30.83	32.92	32.80	33.42	31.00	31.00	33.50
8	30.56	34.69	33.26	33.50	30.72	34.06	31.57	33.62

Figure 3C: Effects of Lithium on swimming speed: Lithium 0.5 mg/kg

Rat	Trial 1	Trial 2	Trial 3	Trial 4	Trial 5	Trial 6	Trial 7	Trial 8
1	29.38	30.72	31.12	33.62	33.85	30.45	35.00	32.22
2	30.81	30.05	32.25	33.38	34.30	34.35	34.85	31.90
3	30.24	33.55	31.44	33.31	33.52	30.75	34.87	31.50
4	29.81	31.20	35.15	33.45	33.60	33.71	28.75	29.20
5	28.93	31.14	33.07	32.42	31.72	33.93	29.15	31.66
6	29.66	31.56	30.64	32.60	35.50	29.90	32.00	30.09
7	30.17	29.31	31.54	35.31	34.21	29.55	34.42	31.22
8	30.48	29.84	34.28	34.17	35.04	35.07	29.25	31.00

Figure 3C: Effects of Lithium on swimming speed: Lithium 1 mg/kg

Rat	Trial 1	Trial 2	Trial 3	Trial 4	Trial 5	Trial 6	Trial 7	Trial 8
1	29.65	29.50	30.65	33.31	33.47	34.14	31.33	30.71
2	29.57	33.94	33.76	31.19	26.55	32.66	27.83	33.33
3	29.08	33.36	30.60	31.78	26.94	32.57	28.28	30.50
4	29.51	30.88	31.51	32.80	30.33	33.25	29.92	32.14
5	29.26	30.00	30.82	32.44	32.22	31.18	27.30	32.63
6	30.25	32.35	30.93	35.05	32.33	29.66	30.30	33.50
7	29.85	29.51	31.75	35.05	33.46	31.87	29.00	30.12
8	29.61	31.79	31.73	32.62	32.12	34.53	31.00	32.25

Figure 3C: Effects of Lithium on swimming speed: Lithium 5 mg/kg

EXCLI Journal 2018;17:916-934 – ISSN 1611-2156
Received: July 07, 2018, accepted: August 27, 2018, published: September 20, 2018

Figure 3C: Effects of ACPA 0.001 µg/rat + Lithium on swimming speed

Rat	Trial 1	Trial 2	Trial 3	Trial 4	Trial 5	Trial 6	Trial 7	Trial 8
1	29.06	30.60	30.59	33.34	32.06	32.40	33.13	35.05
2	31.19	31.57	31.52	32.75	32.51	32.50	35.05	32.90
3	29.51	30.52	30.71	32.84	31.39	31.77	33.22	35.68
4	30.17	29.76	30.60	31.41	31.68	32.16	31.90	34.50
5	30.08	31.41	33.00	33.00	32.50	32.00	32.90	35.22
6	31.21	31.22	30.40	33.03	31.83	32.07	31.92	34.45
7	30.96	31.18	31.37	32.56	31.71	32.40	31.29	33.31
8	29.31	29.40	31.02	32.03	32.42	31.03	32.32	34.76

Figure 3C: Effects of ACPA 0.001 µg/rat + Lithium on swimming speed:
ACPA 0.001 µg/rat + Saline

Rat	Trial 1	Trial 2	Trial 3	Trial 4	Trial 5	Trial 6	Trial 7	Trial 8
1	31.26	30.15	33.27	33.70	34.84	31.61	33.42	35.66
2	29.35	29.25	31.62	33.52	34.40	33.50	31.50	35.85
3	29.10	31.11	31.94	35.38	35.05	31.07	35.16	28.75
4	29.81	30.18	32.13	33.25	33.54	31.92	35.10	35.71
5	30.52	30.87	33.25	33.64	33.73	32.68	33.36	35.87
6	29.85	30.84	33.09	34.21	30.61	30.18	35.00	32.00
7	31.00	31.01	33.32	34.04	33.94	31.21	31.00	31.00
8	30.32	32.11	33.34	34.61	31.78	31.45	36.50	31.20

Figure 3C: Effects of ACPA 0.001 µg/rat + Lithium on swimming speed:
ACPA 0.001 µg/rat + Lithium 0.5 mg/kg

Rat	Trial 1	Trial 2	Trial 3	Trial 4	Trial 5	Trial 6	Trial 7	Trial 8
1	29.35	29.97	32.93	35.76	34.26	32.46	32.50	34.00
2	29.23	31.26	32.92	33.20	33.05	32.50	33.44	33.14
3	30.10	31.45	32.82	33.85	34.05	32.56	32.55	35.16
4	31.17	32.62	33.33	33.72	34.21	34.54	30.92	34.83
5	29.86	30.33	34.77	34.55	34.87	33.81	32.30	29.66
6	30.87	31.41	32.45	34.42	34.29	31.71	34.57	29.90
7	31.58	30.16	31.14	33.44	34.36	33.94	31.46	30.18
8	29.61	29.23	30.59	33.39	34.33	31.58	27.87	34.28

Figure 3C: Effects of ACPA 0.001 µg/rat + Lithium on swimming speed:
ACPA 0.001 µg/rat + Lithium 1 mg/kg

Rat	Trial 1	Trial 2	Trial 3	Trial 4	Trial 5	Trial 6	Trial 7	Trial 8
1	30.20	31.29	32.40	35.33	35.10	31.60	31.20	33.36
2	29.70	26.15	33.28	35.59	32.64	31.27	32.27	28.37
3	30.19	33.02	32.68	33.35	35.04	36.40	31.73	32.09
4	31.39	31.56	32.93	33.75	31.81	31.77	33.36	31.20
5	30.52	31.51	32.00	34.31	34.69	32.12	28.44	30.88
6	29.45	31.12	33.77	36.14	30.81	31.55	29.37	33.42
7	29.68	31.55	35.03	33.08	30.94	31.93	30.00	34.00
8	31.15	31.54	32.48	34.11	31.28	32.38	32.60	32.00

Figure 3C: Effects of ACPA 0.001 µg/rat + Lithium on swimming speed:
ACPA 0.001 µg/rat + Lithium 5 mg/kg

EXCLI Journal 2018;17:916-934 – ISSN 1611-2156
Received: July 07, 2018, accepted: August 27, 2018, published: September 20, 2018

Figure 3C: Effects of AM251 1 ng/rat + Lithium on swimming speed

Rat	Trial 1	Trial 2	Trial 3	Trial 4	Trial 5	Trial 6	Trial 7	Trial 8
1	29.24	31.03	31.12	33.06	32.30	32.69	32.77	34.35
2	29.01	30.86	31.15	32.68	33.11	32.36	33.04	33.50
3	30.76	30.72	31.21	31.54	32.91	32.17	33.22	33.42
4	30.55	31.18	33.87	32.84	32.92	31.89	31.70	33.50
5	29.26	30.71	31.20	31.56	32.53	32.41	34.10	33.05
6	30.67	30.77	30.20	31.16	31.93	33.72	33.12	32.44
7	29.33	30.36	30.85	33.33	31.41	32.72	34.05	29.20
8	31.09	29.71	29.95	32.97	31.68	32.58	31.36	32.10

Figure 3C: Effects of AM251 1 ng/rat + Lithium on swimming speed:
AM251 1 ng/rat + Saline

Rat	Trial 1	Trial 2	Trial 3	Trial 4	Trial 5	Trial 6	Trial 7	Trial 8
1	29.26	30.82	32.45	35.10	31.31	30.91	33.37	35.33
2	29.80	31.33	31.11	34.90	29.73	32.10	33.33	31.36
3	31.00	31.73	33.46	34.80	30.91	31.36	35.12	31.30
4	30.70	30.60	32.24	34.50	35.63	30.06	34.18	34.00
5	31.08	30.17	31.12	32.55	34.54	29.31	32.58	33.11
6	30.40	31.15	32.17	34.26	29.53	29.00	32.77	39.00
7	29.10	31.31	32.57	32.60	35.60	32.58	32.08	35.16
8	29.67	29.86	30.16	32.03	33.23	30.07	31.36	37.42

Figure 3C: Effects of AM251 1 ng/rat + Lithium on swimming speed:
AM251 1 ng/rat + Lithium 0.5 mg/kg

Rat	Trial 1	Trial 2	Trial 3	Trial 4	Trial 5	Trial 6	Trial 7	Trial 8
1	30.80	30.58	31.36	34.10	33.20	35.05	33.16	37.25
2	31.17	29.96	31.43	34.13	34.64	30.07	32.10	36.14
3	29.38	30.39	32.19	33.88	30.46	29.66	33.44	34.87
4	29.51	30.72	31.62	34.27	33.41	34.45	32.68	33.18
5	30.10	30.49	32.02	31.50	34.19	33.00	30.90	35.00
6	30.06	31.08	32.22	33.26	34.42	30.20	33.42	35.87
7	30.94	31.39	33.07	34.76	33.81	35.41	32.11	37.50
8	30.95	31.04	31.55	32.33	33.88	29.85	31.36	36.83

Figure 3C: Effects of AM251 1 ng/rat + Lithium on swimming speed:
AM251 1 ng/rat + Lithium 1 mg/kg

Rat	Trial 1	Trial 2	Trial 3	Trial 4	Trial 5	Trial 6	Trial 7	Trial 8
1	31.03	32.11	31.97	34.26	33.58	30.18	30.90	35.42
2	31.28	33.62	31.65	33.42	35.17	34.78	32.25	33.45
3	29.61	30.90	32.03	33.59	33.85	33.35	32.09	32.88
4	29.86	31.43	31.40	31.52	34.00	34.52	29.28	32.18
5	30.20	31.07	31.13	35.33	33.17	31.41	34.75	36.00
6	29.26	30.05	38.25	33.66	33.63	32.46	31.22	34.50
7	30.43	31.31	32.45	33.85	34.66	35.10	30.80	34.14
8	30.16	30.90	32.10	33.90	28.86	29.21	31.10	34.42

Figure 3C: Effects of AM251 1 ng/rat + Lithium on swimming speed:
AM251 1 ng/rat + Lithium 5 mg/kg

EXCLI Journal 2018;17:916-934 – ISSN 1611-2156
Received: July 07, 2018, accepted: August 27, 2018, published: September 20, 2018

Raw Data

Escape latency (Time): sec
Traveled distance: cm
Swimming speed: cm/sec

Probes

Supplementary Tables "4" for Figure 4:
Effects of different doses of all drugs (ACPA, AM251 and lithium) on spatial memory retrieval were shown. Twenty-four hours after training, all animals (eight rats in each group), were trained for the probe test in MWM apparatus.

EXCLI Journal 2018;17:916-934 – ISSN 1611-2156
Received: July 07, 2018, accepted: August 27, 2018, published: September 20, 2018

Figure 4A: Effects of all drugs on escape latency (Time)

Rat	Saline	ACPA 0.001 µg/rat	ACPA 0.01 µg/rat	ACPA 1 µg/rat
1	**26**	**16**	**16**	**13**
2	**24**	**29**	**9**	**14**
3	**31**	**24**	**18**	**6**
4	**18**	**20**	**14**	**10**
5	**19**	**19**	**12**	**14**
6	**28**	**20**	**15**	**15**
7	**19**	**20**	**14**	**12**
8	**20**	**21**	**15**	**10**

Figure 4A: Effects of ACPA on escape latency (Time)

Rat	Saline	AM251 1 ng/rat	AM251 10 ng/rat	AM251 100 ng/rat
1	**26**	**19**	**19**	**23**
2	**24**	**22**	**21**	**25**
3	**31**	**20**	**21**	**23**
4	**18**	**30**	**20**	**22**
5	**19**	**21**	**24**	**24**
6	**28**	**22**	**22**	**19**
7	**19**	**20**	**24**	**22**
8	**20**	**18**	**22**	**19**

Figure 4A: Effects of AM251 on escape latency (Time)

Rat	Saline	Lithium 0.5 mg/kg	Lithium 1 mg/kg	Lithium 5 mg/kg
1	**26**	**20**	**19**	**16**
2	**24**	**24**	**20**	**14**
3	**31**	**22**	**27**	**15**
4	**18**	**23**	**24**	**15**
5	**19**	**18**	**25**	**12**
6	**28**	**28**	**21**	**13**
7	**19**	**27**	**26**	**14**
8	**20**	**19**	**22**	**12**

Figure 4A: Effects of Lithium on escape latency (Time)

EXCLI Journal 2018;17:916-934 – ISSN 1611-2156
Received: July 07, 2018, accepted: August 27, 2018, published: September 20, 2018

Figure 4B: Effects of all drugs on traveled distance

Rat	Saline	ACPA 0.001 µg/rat	ACPA 0.01 µg/rat	ACPA 1 µg/rat
1	**892**	**636**	**622**	**414**
2	**851**	**991**	**385**	**459**
3	**1020**	**868**	**747**	**222**
4	**723**	**809**	**574**	**402**
5	**783**	**768**	**430**	**536**
6	**952**	**798**	**577**	**582**
7	**775**	**814**	**562**	**436**
8	**792**	**829**	**587**	**387**

Figure 4B: Effects of ACPA on traveled distance

Rat	Saline	AM251 1 ng/rat	AM251 10 ng/rat	AM251 100 ng/rat
1	**892**	**782**	**798**	**873**
2	**851**	**851**	**810**	**892**
3	**1020**	**823**	**827**	**855**
4	**723**	**1009**	**809**	**836**
5	**783**	**812**	**842**	**878**
6	**952**	**852**	**859**	**810**
7	**775**	**820**	**867**	**848**
8	**792**	**798**	**852**	**795**

Figure 4B: Effects of AM251 on traveled distance

Rat	Saline	Lithium 0.5 mg/kg	Lithium 1 mg/kg	Lithium 5 mg/kg
1	**892**	**814**	**782**	**623**
2	**851**	**864**	**800**	**528**
3	**1020**	**820**	**975**	**582**
4	**723**	**842**	**892**	**592**
5	**783**	**784**	**912**	**428**
6	**952**	**982**	**823**	**432**
7	**775**	**962**	**972**	**487**
8	**792**	**794**	**824**	**440**

Figure 4B: Effects of Lithium on traveled distance

EXCLI Journal 2018;17:916-934 – ISSN 1611-2156
Received: July 07, 2018, accepted: August 27, 2018, published: September 20, 2018

Raw Data

Escape latency (Time): sec
Traveled distance: cm
Swimming speed: cm/sec

Probes

Supplementary Tables “5” for Figure 5:
Effects of interaction between cannabinoid drugs and lithium on spatial memory retrieval were shown. Twenty-four hours after training, all animals (eight rats in each group), were trained for the probe test in MWM apparatus.

EXCLI Journal 2018;17:916-934 – ISSN 1611-2156
Received: July 07, 2018, accepted: August 27, 2018, published: September 20, 2018

Figure 5A: Effects of interaction between cannabinoid drugs and lithium on escape latency (Time)

Rat	Saline	Lithium 0.5 mg/kg	Lithium 1 mg/kg	Lithium 5 mg/kg
1	**26**	**20**	**19**	**16**
2	**24**	**24**	**20**	**14**
3	**31**	**22**	**27**	**15**
4	**18**	**23**	**24**	**15**
5	**19**	**18**	**25**	**12**
6	**28**	**28**	**21**	**13**
7	**19**	**27**	**26**	**14**
8	**20**	**19**	**22**	**12**

Figure 5A: Effects of Lithium on escape latency (Time)

Rat	Saline	Lithium 0.5 mg/kg	Lithium 1 mg/kg	Lithium 5 mg/kg
1	**16**	**12**	**14**	**16**
2	**29**	**14**	**12**	**19**
3	**24**	**12**	**15**	**22**
4	**20**	**14**	**16**	**15**
5	**19**	**16**	**11**	**21**
6	**20**	**13**	**13**	**21**
7	**20**	**15**	**15**	**19**
8	**21**	**12**	**12**	**18**

Figure 5A: Effects of ACPA 0.001 µg/rat + Lithium on escape latency (Time)

Rat	Saline	Lithium 0.5 mg/kg	Lithium 1 mg/kg	Lithium 5 mg/kg
1	**19**	**14**	**6**	**19**
2	**22**	**16**	**11**	**28**
3	**20**	**9**	**12**	**20**
4	**30**	**13**	**12**	**29**
5	**21**	**12**	**16**	**17**
6	**22**	**17**	**12**	**27**
7	**20**	**8**	**12**	**21**
8	**18**	**11**	**11**	**25**

Figure 5A: Effects of AM251 1 ng/rat + Lithium on escape latency (Time)

Figure 5B: Effects of interaction between cannabinoid drugs and lithium on traveled distance

Rat	Saline	Lithium 0.5 mg/kg	Lithium 1 mg/kg	Lithium 5 mg/kg
1	**892**	**814**	**782**	**623**
2	**851**	**864**	**800**	**528**
3	**1020**	**820**	**975**	**582**
4	**723**	**842**	**892**	**592**
5	**783**	**784**	**912**	**428**
6	**952**	**982**	**823**	**432**
7	**775**	**962**	**972**	**487**
8	**792**	**794**	**824**	**440**

Figure 5B: Effects of Lithium on traveled distance

Rat	Saline	Lithium 0.5 mg/kg	Lithium 1 mg/kg	Lithium 5 mg/kg
1	**636**	**416**	**543**	**602**
2	**991**	**571**	**408**	**789**
3	**868**	**421**	**618**	**842**
4	**809**	**584**	**645**	**578**
5	**768**	**625**	**402**	**802**
6	**798**	**472**	**489**	**834**
7	**814**	**575**	**607**	**793**
8	**829**	**438**	**434**	**783**

Figure 5B: Effects of ACPA 0.001 µg/rat + Lithium on traveled distance

Rat	Saline	Lithium 0.5 mg/kg	Lithium 1 mg/kg	Lithium 5 mg/kg
1	**782**	**514**	**269**	**799**
2	**851**	**598**	**396**	**987**
3	**823**	**389**	**419**	**841**
4	**1009**	**461**	**405**	**962**
5	**812**	**424**	**628**	**757**
6	**852**	**752**	**443**	**921**
7	**820**	**361**	**425**	**816**
8	**798**	**404**	**421**	**902**

Figure 5B: Effects of AM251 1 ng/rat + Lithium on traveled distance
